# Effect of adjuvant therapy with compound danshen drip pill on inflammatory factors and cardiac function after percutaneous coronary intervention for acute myocardial infarction: a systematic review and meta-analysis

**DOI:** 10.3389/fphar.2024.1345897

**Published:** 2024-04-16

**Authors:** Genhao Fan, Menglin Liu, Huanhuan Song, Yongxia Wang

**Affiliations:** ^1^ The First Affiliated Hospital of Zhengzhou University, Zhengzhou, Henan, China; ^2^ The First Affiliated Hospital of Henan University of Chinese Medicine, Zhengzhou, Henan, China

**Keywords:** Acute myocardial infarction, Compound danshen drip pill, Meta-analysis, Systematic review, Cardiac function

## Abstract

**Objectives:** The purpose of the study was to comprehensively evaluate efficacy and safety of CDDP in patients with AMI undergoing PCI.

**Methods:** A computerised search was conducted on the CNKI, WF, VIP, CBM, PubMed, Embase, Web of Science, and Cochrane Library databases for RCTs of CDDP adjuvant therapy for AMI up to May 2023. STATA 17.0 was used to perform meta-analyses, sensitivity analyses, subgroup analyses, meta-regression, and publication bias assessments. TSA 0.9.5.10 Beta was used for trial sequential analysis (TSA). Evidence confidence of meta results was evaluated by GRADE (Grading of Recommendations Assessment, Development and Evaluation) according to the instructions.

**Results:** The results of the meta-analysis showed that CDDP combined with conventional western treatment (CWT) was superior to CWT in increasing LVEF and TCER and decreasing LVEDD, hs-CRP, IL-6 and TNF-α. The quality of evidence for TCER was moderate, LVEF, LVEDD, IL-6, and TNF-α were low. The TSA results showed that the total number of samples collected in this study met the requirements for meta-analysis and excluded the possibility of false positives, further confirming the efficacy of CDDP for the treatment of AMI undergoing PCI.

**Conclusion:** Adjuvant treatment of AMI with CDDP has shown exciting and safe benefits in improving cardiac function and reducing inflammatory response in patients with AMI undergoing PCI, but the quality of some of the included studies was poor, and the results should be interpreted with caution until further confirmation by well-designed RCTs.

Systematic Review Registration: [https://www.crd.york.ac.uk/PROSPERO/#recordDetails], identifier [CRD42023453293].

## Highlights

1. This study is the first trial sequential analysis and meta-analysis of the treatment of AMI undergoing PCI with CDDP.
2. CDDP has been shown to have a positive effect on improving cardiac function and suppressing inflammation.
3. The complementary or alternative therapies combined with conventional medicine are beneficial to the AMI undergoing PCI.

## 1 Introduction

AMI is myocardial necrosis caused by acute and persistent ischemia and hypoxia of the coronary arteries, with clinical manifestations of severe and persistent retrosternal pain, increased myocardial enzyme activity, and progressive changes in the electrocardiogram, which can involve respiratory, digestive, and cardiovascular systems and be accompanied by cardiac failure, arrhythmia, and cardiogenic shock, which can seriously endanger the lives of patients (Bhatt et al., 2022). Annually, about 7.2 million people in the United States are affected by AMI, with patients over the age of 75 accounting for approximately 30%–40% of hospital admissions (Damluji et al., 2023). Since 2005, the mortality rate for AMI patients has increased rapidly, with female patients showing a tendency to have a higher mortality rate than male patients (Shengshou et al., 2019). AMI has a high morbidity and mortality rate, posing a serious threat to human health and life safety, and placing a heavy burden on patients and their families (Roohafza et al., 2023).

AMI can be categorized into ST segment elevation myocardial infarction (STEMI) and non-ST segment elevation myocardial infarction (NSTEMI). This classification is based on the presence or absence of ST segment elevation on electrocardiogram (ECG). STEMI is caused by the complete occlusion of coronary arteries, and ischemia-reperfusion therapy should be implemented as early as possible to open the infarcted vessel, reducing the infarcted area or the extent of myocardial ischemia, decreasing the rate of death, and improving prognosis. For patients with NSTEMI, the GRACE and TIMI risk scores are commonly used in clinical practice to identify their ischemic risk. It is also important to consider early implementation of ischemia-reperfusion therapy for high-risk NSTEMI patients (Ibanez et al., 2018). PCI is currently considered the most effective treatment for STEMI. However, it is important to note that sudden reperfusion of ischemic myocardium can cause ischemia-reperfusion injury (IRI) (Yellon and Hausenloy, 2007; Hausenloy and Yellon, 2008), IRI-induced cardiac dysfunction comprises systolic dysfunction, reperfusion arrhythmias, endothelial dysfunction, and lethal reperfusion injury (Qin et al., 2009). The mortality rate at 90 days after ischemia-reperfusion can be as high as 5% (Stebbins et al., 2010). Therefore, it is urgent to find effective targeted drugs to inhibit IRI (Rout et al., 2020).

CDDP is a Chinese medicine that combines modern medical technology with traditional Chinese medicine (TCM) theories. It has successfully passed Phase III clinical trials by the U.S. FDA, making it the first proprietary Chinese medicine to do so (Liang et al., 2020). CDDP is mainly concocted from three herbs, *Salviae Miltiorrhizae Radix et Rhizoma*, *Notoginseng Radix*, and *Borneolum Syntheticum*. Pharmacological studies have shown that CDDP has the ability to inhibit oxidative stress, platelet aggregation, and calcium channels, as well as dilate coronary blood vessels (Li, 2018). Currently, there is a growing number of clinical studies on the treatment of AMI. CWT has been found to be effective in treating AMI, but it is also associated with more adverse reactions. The dosage and types of drugs used are also increasing, leading to a corresponding increase in side effects. TCM has distinct advantages in treating cardiovascular diseases due to its lower incidence of side effects and superior long-term effectiveness (Wang et al., 2018). This study aims to comprehensively and systematically evaluate the efficacy and safety of CDDP in treating AMI undergoing PCI, following PRISMA guidelines. The goal is to provide a more reliable evidence-based basis for the clinical use of CDDP.

## 2 Information and methods

### 2.1 Protocol and registration

This systematic review and meta-analysis of RCTs were conducted by PRISMA guidelines (Moher et al., 2009). The protocol studied has been registered with PROSPERO under the registration number CRD42023453293.

### 2.2 Literature sources

A computerised search was conducted on the CNKI, WF, VIP, CBM, PubMed, Embase, Web of Science, and Cochrane Library databases for RCTs of CDDP adjuvant therapy for AMI up to May 2023. There is no restriction on the language of the search using subject terms. The search terms are Compound Danshen Dropping Pill, Danshen Dropping Pill, Acute Myocardial Infarction, Percutaneous Coronary Intervention, PCI, and AMI. Search strategies were provided in Supplementary Material S1.

### 2.3 Inclusion criteria for screening studies

#### 2.3.1 Type of participants (P)

Compliance with the Guidelines for Integrating Chinese and Western Medicine in the Diagnosis and Treatment of Acute Myocardial Infarction or (Zhang, 2018) the American College of Cardiology/American Heart Association (ACC/AHA) diagnostic criteria for AMI (Levine et al., 2016), without restriction on age, gender, or history of smoking or alcohol use.

#### 2.3.2 Type of interventions (I and C)

Control group: Conventional western treatment (CWT), including PCI, antiplatelet, anticoagulation, β-receptor blocker, lipid modulation, angiotensin-converting enzyme inhibitors (ACEI) and angiotensin receptor blockers (ARB), CWT were consistent between groups in each study. Treatment group: CDDP plus CWT.

#### 2.3.3 Type of outcome measures (O)

Primary indicator included left ventricular ejection fraction (LVEF) and total clinical effective rate (TCER) = (obvious + effective) cases/total cases × 100%, obvious: no shift or less than 0.05 mV shift of the ST segment on the ECG, effective: ECG ST segment returns more than 0.05 mV to baseline, ineffective: ECG not improved (Xiaoyu, 2002). Secondary indicator included left ventricular end-diastolic internal diameter (LVEDD), N-terminal pro-B-type natriuretic peptide (NT-proBNP), troponin T (cTnT), creatine kinase isoenzyme (CK-MB), high-sensitivity C-reactive protein (hs-CRP), interleukin-6 (IL-6), tumor necrosis factor-alpha (TNF-α), and adverse reactions/adverse events.

#### 2.3.4 Types of studies (S)

RCTs of CDDP in combination with CWT for AMI.

Exclusion criteria
  Mechanistic studies, reviews, lessons learned, and case reports.
  Duplicate publications.
  Incomplete documentation

### 2.4 Literature screening and extraction

Two researchers independently read the full text and extracted relevant information. The extracted information included basic information, intervention methods, risk of bias assessment, relevant outcome indicators and adverse reactions, etc. When the two parties disagreed on the inclusion of the literature, it was referred to a third party for discussion and judgment.

### 2.5 Literature quality assessment

The Cochrane risk-of-bias tool for randomized trials was used to assess each study’s risk of bias (Sterne et al., 2019). This tool comprises a five-item checklist: 1) randomization process; 2) deviations from the intended interventions; 3) missing outcome data; 4) measurement of the outcome; and 5) selection of the reported result. The risk of each domain was examined as “low”, “high” and “some concerns”.

### 2.6 Statistical processing

The included data were statistically analyzed using STATA 17.0. Continuous data was pooled with Std mean difference (SMD) and 95% confidence interval (CI), dichotomous data was pooled with relative risk (RR) and 95% CI. Heterogeneity was judged based on the results of the I^2^ test; when I^2^<50%, a fixed-effects model was used, and when I^2^ ≥ 50% indicated that inter-study heterogeneity was significant, so the reasons for heterogeneity were analyzed. First, the raw data were checked for correctness, and second, if heterogeneity was attributed to treatment duration, sample size, publication time, etc., meta-regression and subgroup analyses could be used to investigate the sources of heterogeneity. Sensitivity analysis evaluates the robustness and reliability of the results. If an outcome had more than 10 articles, a funnel plot was analysed for publication bias and publication bias was evaluated using Egger’s test with STATA 12.0.

### 2.7 Trial sequential analysis (TSA) and evidence confidence

Trial sequential analysis (TSA) was performed using TSA 0.9.5.10 Beta software (Greco and Capodanno, 2024). Evidence confidence of meta results was evaluated by GRADE (Grading of Recommendations Assessment, Development and Evaluation) according to the instructions (Brozek et al., 2009).

## 3 Results

### 3.1 Literature search

**FIGURE 1:**
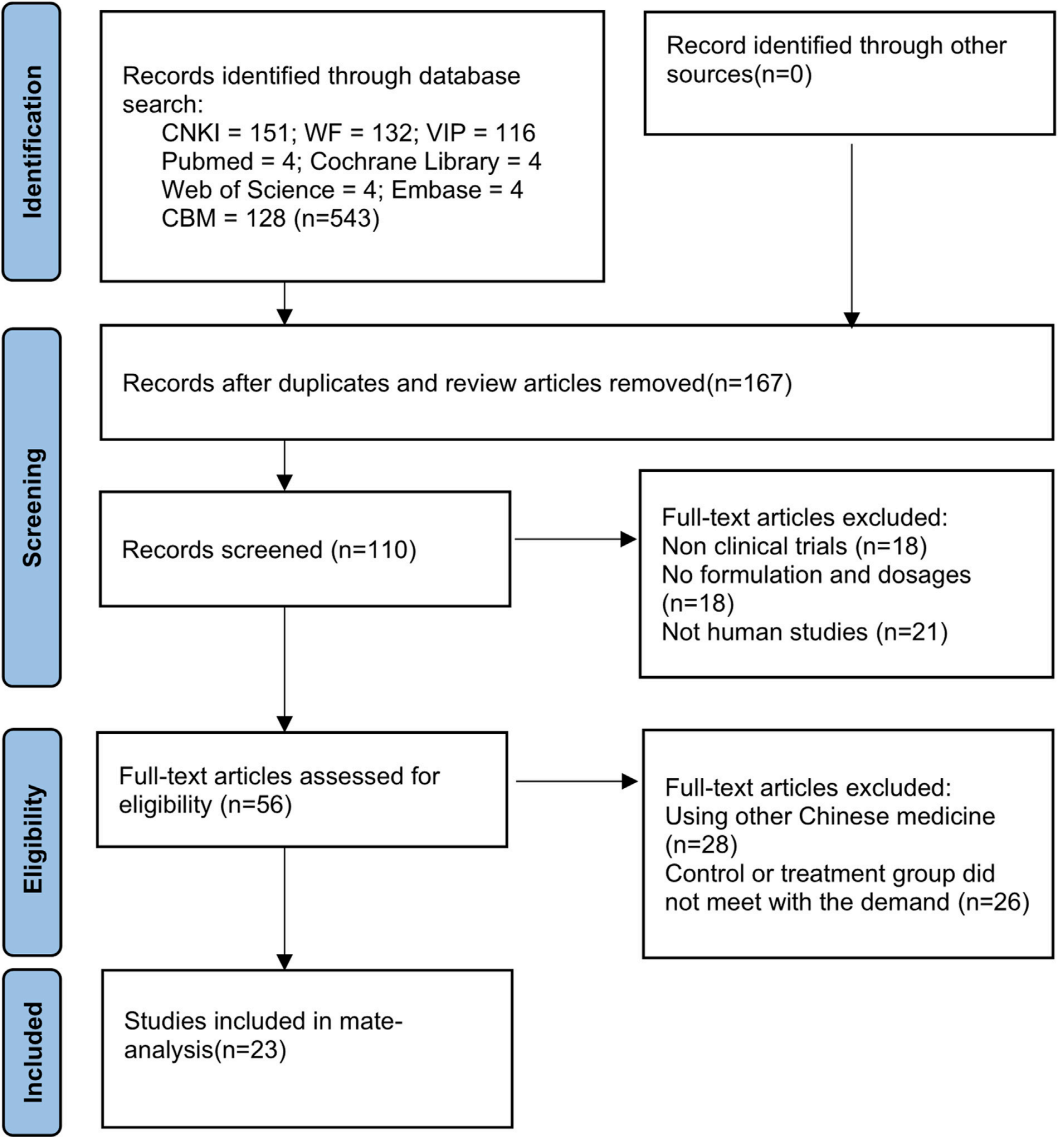
Flow diagram for the study selection process.

A total of 543 studies were searched. The retrieved titles were imported into EndNote X9, and 23 studies (Li et al., 2010; Li et al., 2011; Lin, 2011; Fang et al., 2017; Pan et al., 2017; Zhang, 2017; Li, 2018; Guihong et al., 2018; Hong et al., 2018; Ji et al., 2019; Li et al., 2019; Liu and Zhang, 2019; Shi, 2019; Su, 2019; Wenhui, 2019; Xiang, 2019; Xu et al., 2019; Zhao et al., 2019; Jian, 2020; Li, 2020; Niu, 2020; Shi, 2020; Chen, 2023) were finally included after checking and screening (Figure 1).

### 3.2 Study characteristics

A total of 23 studies (Li et al., 2010; Li et al., 2011; Lin, 2011; Fang et al., 2017; Pan et al., 2017; Zhang, 2017; Li, 2018; Guihong et al., 2018; Hong et al., 2018; Ji et al., 2019; Li et al., 2019; Liu and Zhang, 2019; Shi, 2019; Su, 2019; Wenhui, 2019; Xiang, 2019; Xu et al., 2019; Zhao et al., 2019; Jian, 2020; Li, 2020; Niu, 2020; Shi, 2020; Chen, 2023) were included in this meta-analysis, all of which were conducted in China and involved 2,732 AMI patients, including 1,377 in the treatment group and 1,355 in the control group. In all studies, there were no statistical differences between the experimental and control groups in terms of age and sample size. LVEF was reported in 19 studies (Li et al., 2010; Li et al., 2011; Lin, 2011; Pan et al., 2017; Zhang, 2017; Guihong et al., 2018; Ji et al., 2019; Li et al., 2019; Liu and Zhang, 2019; Shi, 2019; Su, 2019; Wenhui, 2019; Xiang, 2019; Xu et al., 2019; Zhao et al., 2019; Jian, 2020; Li, 2020; Niu, 2020; Shi, 2020), TCER in 6 studies (Guihong et al., 2018; Li et al., 2019; Xiang, 2019; Xu et al., 2019; Zhao et al., 2019; Shi, 2020), LVEDD in 9 studies (Fang et al., 2017; Ji et al., 2019; Li et al., 2019; Wenhui, 2019; Xiang, 2019; Xu et al., 2019; Jian, 2020; Li, 2020; Niu, 2020), NT-proBNP in 3 studies (Fang et al., 2017; Li et al., 2019; Shi, 2020), cTnT in 2 studies (Ji et al., 2019; Chen, 2023), CK-MB in 3 studies (Ji et al., 2019; Li et al., 2019; Zhao et al., 2019), hs-CRP in 4 studies (Li, 2018; Shi, 2019; Zhao et al., 2019; Niu, 2020), IL-6 in 6 studies (Fang et al., 2017; Pan et al., 2017; Li, 2018; Hong et al., 2018; Zhao et al., 2019; Niu, 2020), TNF-α in 5 studies (Pan et al., 2017; Li, 2018; Hong et al., 2018; Shi, 2019; Niu, 2020), and adverse reactions/adverse events in 11 studies (Li et al., 2010; Li, 2018; Guihong et al., 2018; Ji et al., 2019; Li et al., 2019; Liu and Zhang, 2019; Wenhui, 2019; Zhao et al., 2019; Jian, 2020; Niu, 2020; Shi, 2020) (Table 1).

**TABLE 1:** Literature features.

References	Sample size		Age (years)		Treatment duration	Interventions		Outcome measure
	T	C	T	C		T	C	
Chen (2023)	53	53	62.12 ± 7.86	63.15 ± 6.51	4 weeks	CWT + CDDP, 10 pills, 3 times/day	CWT	cTnT
Niu (2020)	35	35	70.22 ± 5.12	68.51 ± 3.15	4 weeks	CWT + CDDP, 10 pills, 3 times/day	CWT	LVEF, LVEDD, hs-CRP, IL-6, TNF-α, ADR/AE
Shi (2020)	42	42	57.5 ± 2.3	56.9 ± 2.4	1 month	CWT + CDDP, 10 pills, 3 times/day	CWT	LVEF, NT-proBNP, TCER, ADR/AE
Li (2020)	46	46	66.74 ± 5.23	66.98 ± 5.41	4 weeks	CWT + CDDP, 10 pills, 3 times/day	CWT	LVEF, LVEDD
Chen 2020	51	51	69.07 ± 6.13	68.32 ± 5.89	3 months	CWT + CDDP, 10 pills, 3 times/day	CWT	LVEF, LVEDD, ADR/AE
Xu et al. (2019)	40	40	56.85 ± 6.71	55.92 ± 6.64	24 weeks	CWT + CDDP, 10 pills, 3 times/day	CWT	LVEF, LVEDD, TCER
Fu 2019	61	61	63.92 ± 3.56	63.64 ± 3.64	12 weeks	CWT + CDDP, 10 pills, 3 times/day	CWT	LVEF, LVEDD, ADR/AE
Li et al. (2019)	45	45	63.2 ± 8.5	62.95 ± 9.48	2 weeks	CWT + CDDP, 10 pills, 3 times/day	CWT	LVEF, LVEDD, NT-proBNP, CK-MB, TCER, ADR/AE
Su (2019)	75	75	54.1 ± 11.3	53.2 ± 11.6	2 months	CWT + CDDP, 10 pills, 3 times/day	CWT	LVEF
JI et al. (2019)	67	69	-	-	1 week	CWT + CDDP, 10 pills, 3 times/day	CWT	LVEF, LVEDD, cTnT, CK-MB, ADR/AE
Liu and Zhang (2019)	40	40	65 ± 2.2	64 ± 2	6 months	CWT + CDDP, 10 pills, 3 times/day	CWT	LVEF, ADR/AE
Zhao et al. (2019)	52	51	54.71 ± 2.95	55.02 ± 3.29	2 weeks	CWT + CDDP, 10 pills, 3 times/day	CWT	LVEF, CK-MB, TCER, hs-CRP, IL-6, ADR/AE
Xiang (2019)	20	20	56.7 ± 7.5	56.9 ± 7.9	15 days	CWT + CDDP, 10 pills, 3 times/day	CWT	LVEF, LVEDD, TCER
Shi (2019)	50	50	60.13 ± 4.21	60.20 ± 4.15	6 months	CWT + CDDP, 10 pills, 3 times/day	CWT	LVEF, hs-CRP, TNF-α
Li (2018a)	40	40	65.18 ± 5.69	64.38 ± 5.07	3 months	CWT + CDDP, 10 pills, 3 times/day	CWT	hs-CRP, IL-6, TNF-α, ADR/AE
Gong 2018	60	60	56.70 ± 7.48	56.95 ± 7.92	15 days	CWT + CDDP, 10 pills, 3 times/day	CWT	LVEF, TCER, ADR/AE
Chen 2018	60	60	66.53 ± 2.3	65.62 ± 3.6	4 weeks	CWT + CDDP, 10 pills, 3 times/day	CWT	IL-6, TNF-α
Zhang (2017)	76	84	58.9 ± 9.2	58.5 ± 8.1	3 months	CWT + CDDP, 10 pills, 3 times/day	CWT	LVEF
Pan 2017	66	58	59.46 ± 7.85	55.02 ± 8.37	30 days	CWT + CDDP, 10 pills, 3 times/day	CWT	LVEF, IL-6, TNF-α
Fang et al. (2017)	60	60	64.52 ± 1.03	63.95 ± 0.89	3 months	CWT + CDDP, 10 pills, 3 times/day	CWT	LVEDD, NT-proBNP, IL-6
Lin (2011)	46	44	-	-	6 weeks	CWT + CDDP, 10 pills, 3 times/day	CWT	LVEF
Li et al. (2011a)	250	250	60.1 ± 9.6	56.7 ± 7.8	30 days	CWT + CDDP, 10 pills, 3 times/day	CWT	LVEF
Li et al. (2010)	42	21	-	-	4 months	CWT + CDDP, 10 pills, 3 times/day	CWT	LVEF, ADR/AE

Note: T: Treatment group; C: Control group; ADR/AE: Adverse Reactions/Adverse Events.

### 3.3 Quality assessment

**FIGURE 2:**
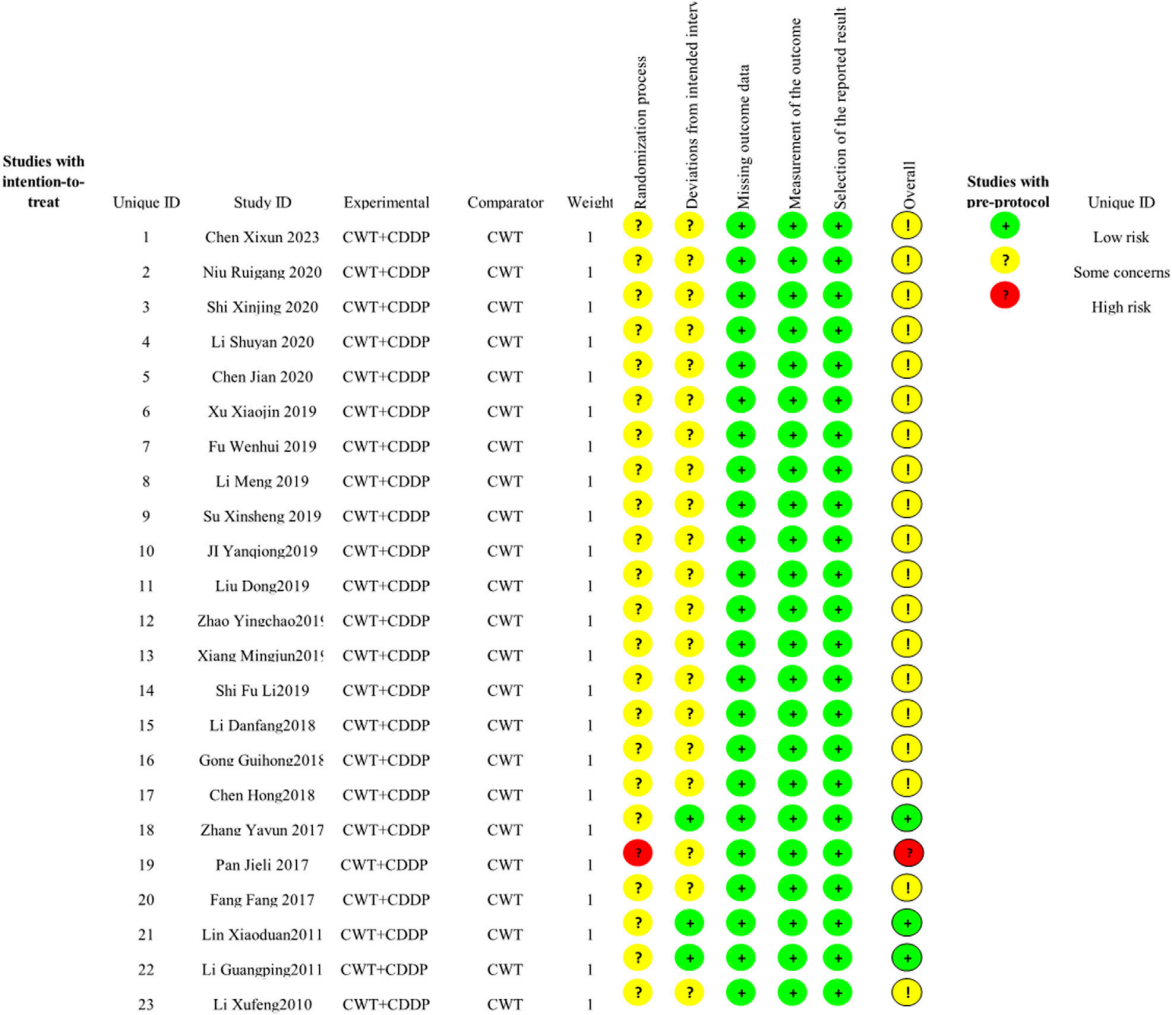
Risk of bias graph.

In the 23 included studies, 13 used a random-number table, and the aspect of whether the order of allocation was randomized was described as “low risk”. One study was randomized using order of presentation, which may have been at risk of bias, described as “high risk”. All studies did not mention allocation hiding, so the randomization process section was listed as “some concern”. Three studies were blinded to subjects and implementers, and no information was described for the other 19 studies. There was no missing data and no selective results in any of the studies, so these sections were described as “low risk” (Figure 2).

## 4 Meta-analysis results

### 4.1 Cardiac function

#### 4.1.1 LVEF

**FIGURE 3:**
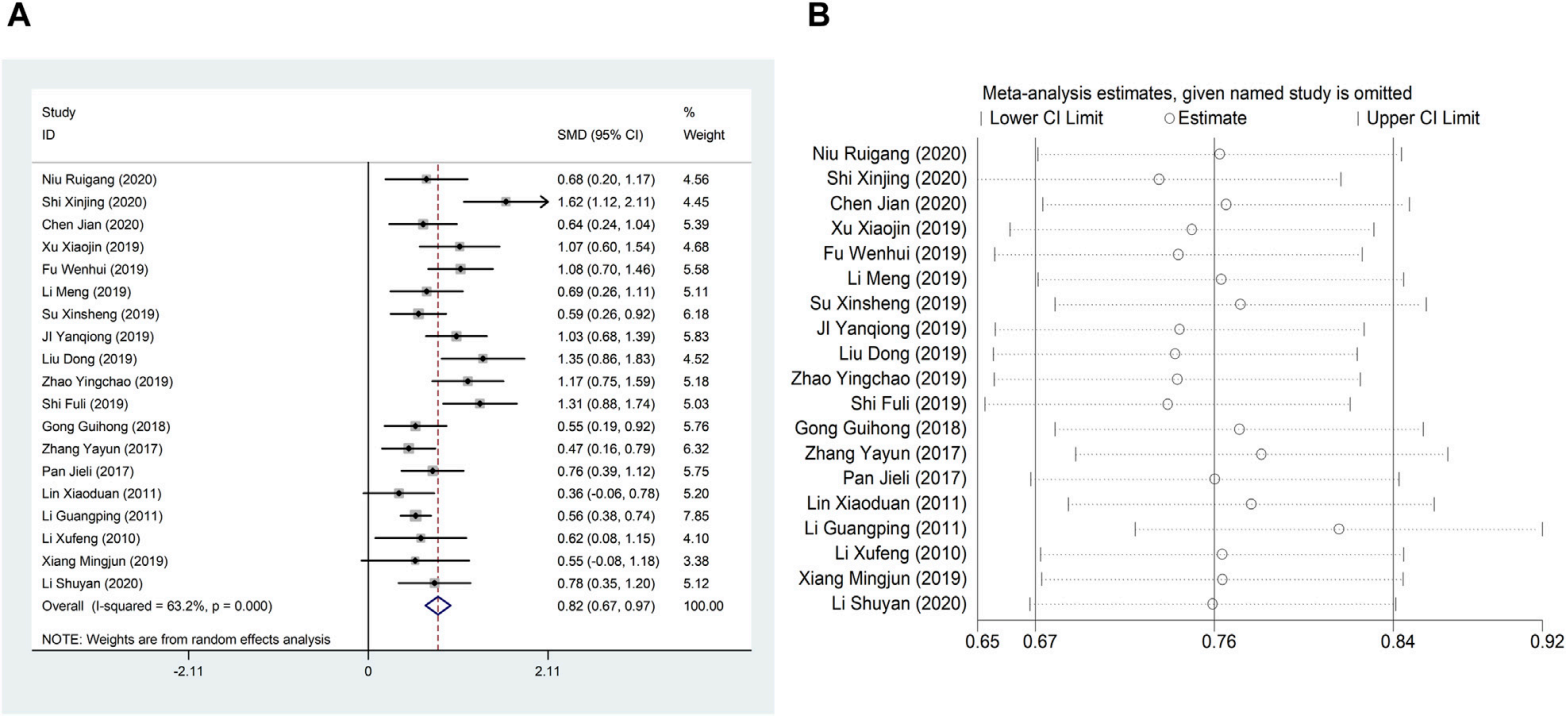
A Forest plot of LVEF; B Sensitivity analysis of LVEF.

Nineteen studies (Li et al., 2010; Li et al., 2011; Lin, 2011; Pan et al., 2017; Zhang, 2017; Guihong et al., 2018; Ji et al., 2019; Li et al., 2019; Liu and Zhang, 2019; Shi, 2019; Su, 2019; Wenhui, 2019; Xiang, 2019; Xu et al., 2019; Zhao et al., 2019; Jian, 2020; Li, 2020; Niu, 2020; Shi, 2020) reported LVEF, and due to the large heterogeneity (I^2^ = 63.2%), so meta-analysis using a random effects model showed statistically significant differences (SMD = 0.82, 95%CI (0.67, 0.97), *p* < 0.0001), and this result indicated that CDDP added to CWT was superior to CWT in improving LVEF (Figure 3A). Because of inter-study heterogeneity, we performed a sensitivity analysis to exclude any study that did not affect the overall estimate of effect (Figure 3B). To further clarify the sources of heterogeneity, we also conducted meta-regression to evaluate the effects of the publication time (Coed. = 0.374, *p* = 0.019), sample size (Coed. = −0.014, *p* = 0.991), and treatment duration (Coed. = −0.219, *p* = 0.856), the results suggest that publication time may be a source of high heterogeneity (Table 2). Subgroup analyses confirmed less heterogeneity for publication dates before 2018. Subgroup analyses also found significantly less heterogeneity in subgroups with a treatment duration of less than 4 weeks (Table 3, Supplementary Material S2).

**TABLE 2:** Meta regression analysis.

Meta regression analysis on the results of LVEF					
_ES	Coefficient	Std. err	t	p > |t|	[95% conf. interval]
Publication time	0.374	0.145	2.580	0.019	.0682516 .6803397
Sample size	−0.060	0.161	−0.370	0.716	−.4004015 .28081
Treatment duration	0.006	0.184	0.030	0.973	−.3812294 .3939737

Meta regression analysis on the results of LVEDD					
_ES	Coefficient	Std. err	t	p > |t|	[95% conf. interval]
Sample size	−0.267	0.280	−0.950	0.373	−.9291272 .3957474
Publication time	1.062	0.245	4.340	0.003	.4828743 1.640549
Treatment duration	−0.315	0.296	−1.070	0.322	−1.015004 .3841212

Meta regression analysis on the results of NT-proBNP					
_ES	Coefficient	Std. err	t	p > |t|	[95% conf. interval]
Sample size	3.463	7.348	0.470	0.720	−89.90199 96.82829
Publication time	−3.463	7.348	−0.470	0.720	−96.8283 89.90199
Treatment duration	−4.597	6.690	−0.690	0.617	−89.60107 80.40689

Meta regression analysis on the results of CK-MB					
_ES	Coefficient	Std. err	t	p > |t|	[95% conf. interval]
Sample size	0.054	1.188	0.050	0.971	−15.03724 15.14599

Meta regression analysis on the results of hs-CRP					
_ES	Coefficient	Std. err	t	p > |t|	[95% conf. interval]
Sample size	−0.453	0.525	−0.860	0.479	−2.711179 1.805803
Treatment duration	0.937	0.286	3.270	0.082	−.2949249 2.168454

Meta regression analysis on the results of IL-6					
_ES	Coefficient	Std. err	t	p > |t|	[95% conf. interval]
Sample size	−3.003	5.100	−0.590	0.588	−17.16378 11.15833
Publication time	−3.661	4.988	−0.730	0.504	−17.50982 10.18752
Treatment duration	−2.628	6.593	−0.400	0.711	−20.93371 15.67704

Meta regression analysis on the results of TNF-α					
_ES	Coefficient	Std. err	t	p > |t|	[95% conf. interval]
Sample size	−1.577	4.066	−0.390	0.724	−14.51854 11.36373
Publication time	−3.342	4.711	−0.710	0.529	−18.33346 11.64948

**TABLE 3:** Subgroup analyses.

	Number	Heterogeneity		SMD (95%CI)		P within group
		I^2^	P			
LVEF						
Sample size						
≥50 cases	10	64.2	*p* = 0.003	0.79 (0.61,0.97)		*p* < 0.0001
<50 cases	9	63.9	*p* = 0.005	0.86 (0.59,1.12)		*p* < 0.0001
publication time						
≥2018	14	56.8	*p* = 0.005	0.93 (0.76,1.10)		*p* < 0.0001
<2018	5	0	*p* = 0.68	0.55 (0.42,0.68)		*p* < 0.0001
treatment duration						
≥4 weeks	14	68.3	*p* < 0.0001	0.82 (0.64,1.00)		*p* < 0.0001
<4 weeks	5	44.1	*p* = 0.128	0.82 (0.67,0.97)		*p* < 0.0001
LVEDD						
Sample size						
≥50 cases	4	84.1	*p* < 0.0001	−1.14 −(−1.63,−0.65)		*p* < 0.0001
<50 cases	5	31.2	*p* = 0.213	−0.86 −(−1.12,−0.60)		*p* < 0.0001
publication time						
≥2018	8	3	*p* = 0.406	−0.87 −(−1.3,−0.72)		*p* < 0.0001
<2018	1			−1.94 −(−2.37,−1.50)		*p* < 0.0001
treatment duration						
≥4 weeks	6	74.5	*p* = 0.001	−1.10 −(−1.45,−0.75)		*p* < 0.0001
<4 weeks	3	59.4	*p* = 0.085	−0.79 −(−1.20,−0.37)		*p* < 0.0001
NT-proBNP						
Sample size						
≥50 cases	1			−1.69 −(−2.11,−1.27)		*p* < 0.0001
<50 cases	2	99.1	*p* < 0.0001	−5.15 −(−13.49,3.18)		*p* = 0.226
publication time						
≥2018	2	99.1	*p* < 0.0001	−5.15 −(−13.49,3.18)		*p* = 0.226
<2018	1			−1.69 −(−2.11,−1.27)		*p* < 0.0001
treatment duration						
≥4 weeks	2	98.9	*p* < 0.0001	−5.53 −(−13.12,2.06)		*p* = 0.153
<4 weeks	1			−0.93 −(−1.37,−0.50)		*p* < 0.0001
CK-MB						
Sample size						
≥50 cases	2	95.5	*p* < 0.0001	−1.06 −(−2.40,0.28)		*p* = 0.120
<50 cases	1			−1.12 −(−1.56,−0.67)		*p* < 0.0001
hs-CRP						
Sample size						
≥50 cases	2	86.9	*p* = 0.006	−1.73 −(−2.63,−0.83)		*p* < 0.0001
<50 cases	2	44.6	*p* = 0.179	−1.26 −(−1.73,−0.78)		*p* < 0.0001
treatment duration						
≥4 weeks	3	0	*p* = 0.404	−1.26 −(−1.53,−0.98)		*p* < 0.0001
<4 weeks	1			−2.19 −(−2.68,−1.70)		*p* < 0.0001
IL-6						
Sample size						
≥50 cases	4	98.5	*p* < 0.0001	−4.00 —(−5.98,−2.02)		*p* < 0.0001
<50 cases	2	15.7	*p* = 0.276	−1.44 −(−1.84,−1.05)		*p* < 0.0001
treatment duration						
≥4 weeks	5	98	*p* < 0.0001	−3.44 −(−5.05,−1.82)		*p* < 0.0001
<4 weeks	1			−1.25 −(−4.22,−1.68)		*p* < 0.0001
publication time						
≥2018	4	98.4	*p* < 0.0001	−4.35 −(−6.66,−2.05)		*p* < 0.0001
<2018	2	78.7	*p* = 0.03	−1.00 −(−1.58,−0.42)		*p* = 0.001
TNF-α						
Sample size						
≥50 cases	3	98.9	*p* < 0.0001	−3.72 −(−6.62,−0.83)		*p* = 0.012
<50 cases	2	95.5	*p* < 0.0001	−2.22 −(−4.25,−0.19)		*p* = 0.001
publication time						
≥2018	4	98.4	*p* < 0.0001	−3.78 −(−6.17,−1.39)		*p* = 0.002
<2018	1			−0.51 (−0.86,−0.15)		*p* = 0.006

#### 4.1.2 LVEDD

**FIGURE 4:**
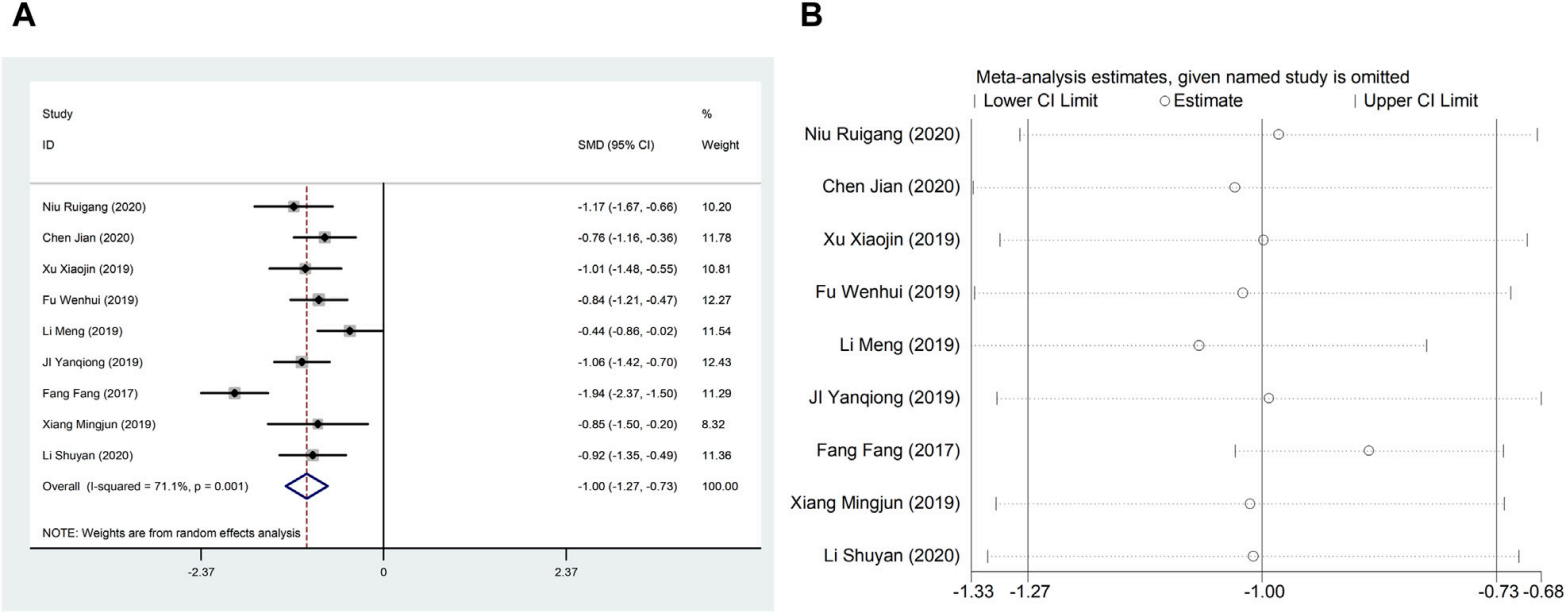
A Forest plot of LVEDD; B Sensitivity analysis of LVEDD.

Nine studies (Fang et al., 2017; Ji et al., 2019; Li et al., 2019; Wenhui, 2019; Xiang, 2019; Xu et al., 2019; Jian, 2020; Li, 2020; Niu, 2020) reported LVEDD with large heterogeneity (I^2^ = 71.1%), so meta-analysis using a random effects model showed statistically significant differences (SMD = −1.00, 95%CI (−1.27, −0.73), *p* < 0.0001), which indicated that CDDP added to CWT was superior to CWT in improving LVEDD (Figure 4A). Because of inter-study heterogeneity, we performed a sensitivity analysis to exclude any study that did not affect the overall estimate of effect (Figure 4B). To further clarify the sources of heterogeneity, we also conducted meta-regression to evaluate the effects of the publication time (Coed. = 1.062, *p* = 0.003), sample size (Coed. = −0.267, *p* = 0.373), and treatment duration (Coed. = −0.315, *p* = 0.322), the results suggest that publication time may be a source of high heterogeneity (Table 2). Subgroup analyses confirmed less heterogeneity in studies published after 2018. This may have been influenced by the rapid development of pharmaceutical technology in recent years and the further optimization of therapeutic options. Subgroup analyses also confirmed better homogeneity with sample sizes less than 50 cases (Table 3, Supplementary Material S2).

#### 4.1.3 NT-proBNP

**FIGURE 5:**
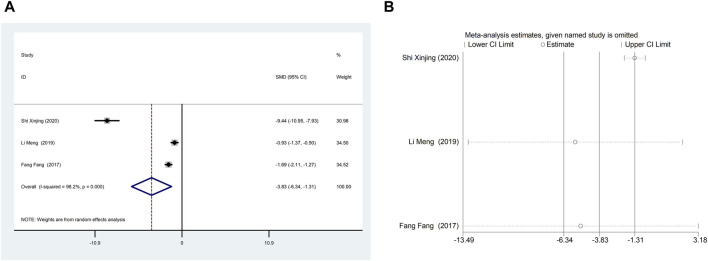
A Forest plot of NT-proBNP; B Sensitivity analysis of NT-proBNP.

Three studies (Fang et al., 2017; Li et al., 2019; Shi, 2020) reported NT-proBNP with large heterogeneity (I^2^ = 98.2%), so meta-analysis using a random effects model showed statistically significant differences (SMD = −3.83, 95%CI (−6.34, −1.31), *p* = 0.003), which indicated that CDDP added to CWT was superior to CWT in improving NT-proBNP (Figure 5A). Because of inter-study heterogeneity, we performed a sensitivity analysis to exclude any study that did not affect the overall estimate of effect (Figure 5B). To further clarify the sources of heterogeneity, we also conducted meta-regression to evaluate the effects of the publication time (Coed. = −3.463, *p* = 0.720), sample size (Coed. = 3.463, *p* = 0.720), and treatment duration (Coed. = −4.597, *p* = 0.617), the results did not reveal the source of heterogeneity among studies (Table 2). Subgroup analyses showed that subgroups with a sample size greater than 50 cases and a treatment duration less than 4 weeks were better at improving NT-proBNP (Table 3, Supplementary Material S2).

#### 4.1.4 cTnT

**FIGURE 6:**
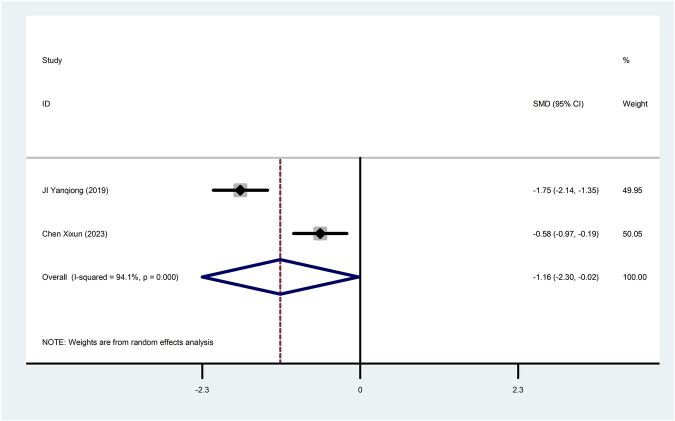
Forest plot of cTnT.

Two studies (Ji et al., 2019; Chen, 2023) reported cTnT with greater heterogeneity (I^2^ = 94.1%), so meta-analysis using a random effects model showed statistically significant differences (SMD = −1.16, 95%CI (−2.30, −0.02), *p* = 0.045), which indicated that CDDP added to CWT was superior to CWT in improving cTnT (Figure 6).

#### 4.1.5 CK-MB

**FIGURE 7:**
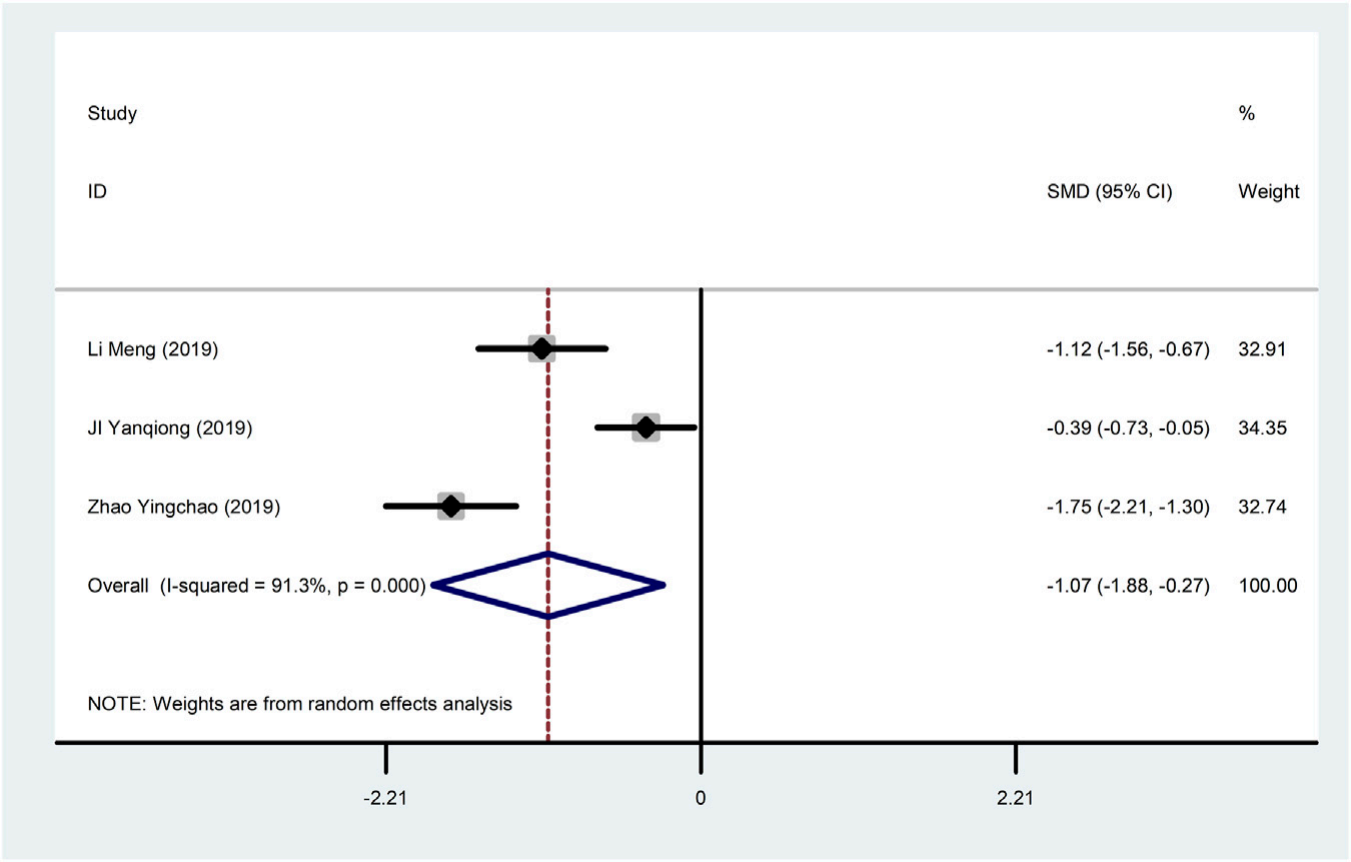
Forest plot of CK-MB.

Three studies (Ji et al., 2019; Li et al., 2019; Zhao et al., 2019) reported CK-MB with large heterogeneity (I^2^ = 91.3%), so meta-analysis using a random effects model showed statistically significant differences (SMD = −1.07, 95%CI (−1.88, −0.27), *p* = 0.009), which indicated that CDDP added to CWT was superior to CWT in improving CK-MB (Figure 7). To further clarify the sources of heterogeneity, we also conducted meta-regression to evaluate the effects of the sample size (Coed. = 0.054, *p* = 0.971), the results did not reveal the source of heterogeneity among studies (Table 2). Subgroup analyses showed that subgroups with sample sizes greater than 50 cases were better at improving CK-MB (Table 3, Supplementary Material S2).

### 4.2 TCER

**FIGURE 8:**
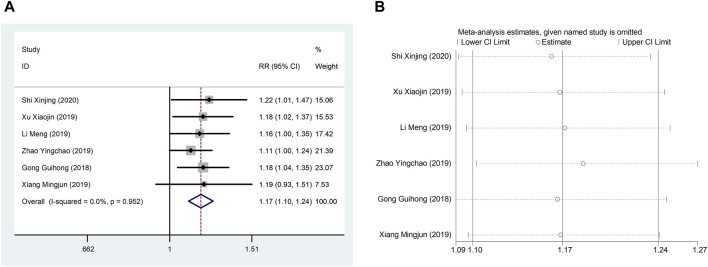
A Forest plot of TCER; B Sensitivity analysis of TCER.

Six studies (Guihong et al., 2018; Li et al., 2019; Xiang, 2019; Xu et al., 2019; Zhao et al., 2019; Shi, 2020) reported TCER with little heterogeneity (I^2^ = 71.1%), therefore, meta-analysis using fixed effect model showed statistically significant between the two groups (RR = 1.17, 95% CI (1.10, 1.24), *p* < 0.0001), which result suggests that CDDP added to CWT is superior to CWT in improving TCER (Figure 8A). Sensitivity analysis showed that excluding any study did not affect the overall estimate of effect (Figure 8B).

### 4.3 Inflammation indicators

#### 4.3.1 hs-CRP

**FIGURE 9:**
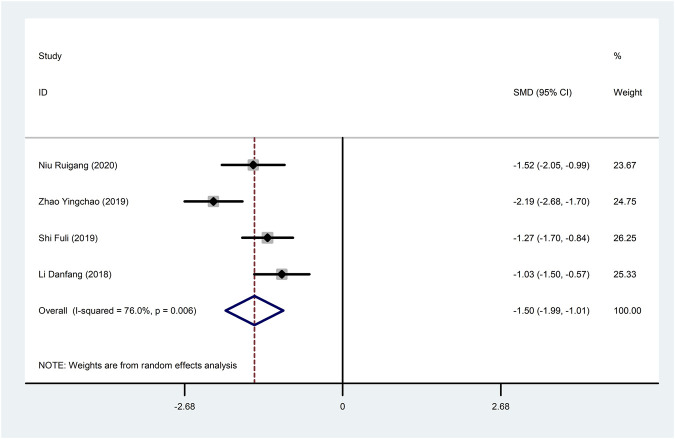
Forest plot of hs-CRP.

Four studies (Li, 2018; Shi, 2019; Zhao et al., 2019; Niu, 2020) reported hs-CRP with large heterogeneity (I^2^ = 76.0%), so meta-analysis using a random effects model showed statistically significant differences (SMD = −1.50, 95%CI (−1.99, −1.01), *p* < 0.0001), which indicated that CDDP added to CWT was superior to CWT in reducing hs-CRP (Figure 9). To further clarify the sources of heterogeneity, we also conducted meta-regression to evaluate the effects of the sample size (Coed. = −0.453, *p* = 0.479) and treatment duration (Coed. = 0.937, *p* = 0.082), the results did not reveal the source of heterogeneity among studies (Table 2). Subgroup analyses showed better homogeneity in subgroups with sample size of less than 50 cases and treatment duration greater than or equal to 4 weeks (Table 3, Supplementary Material S2).

#### 4.3.2 IL-6

**FIGURE 10:**
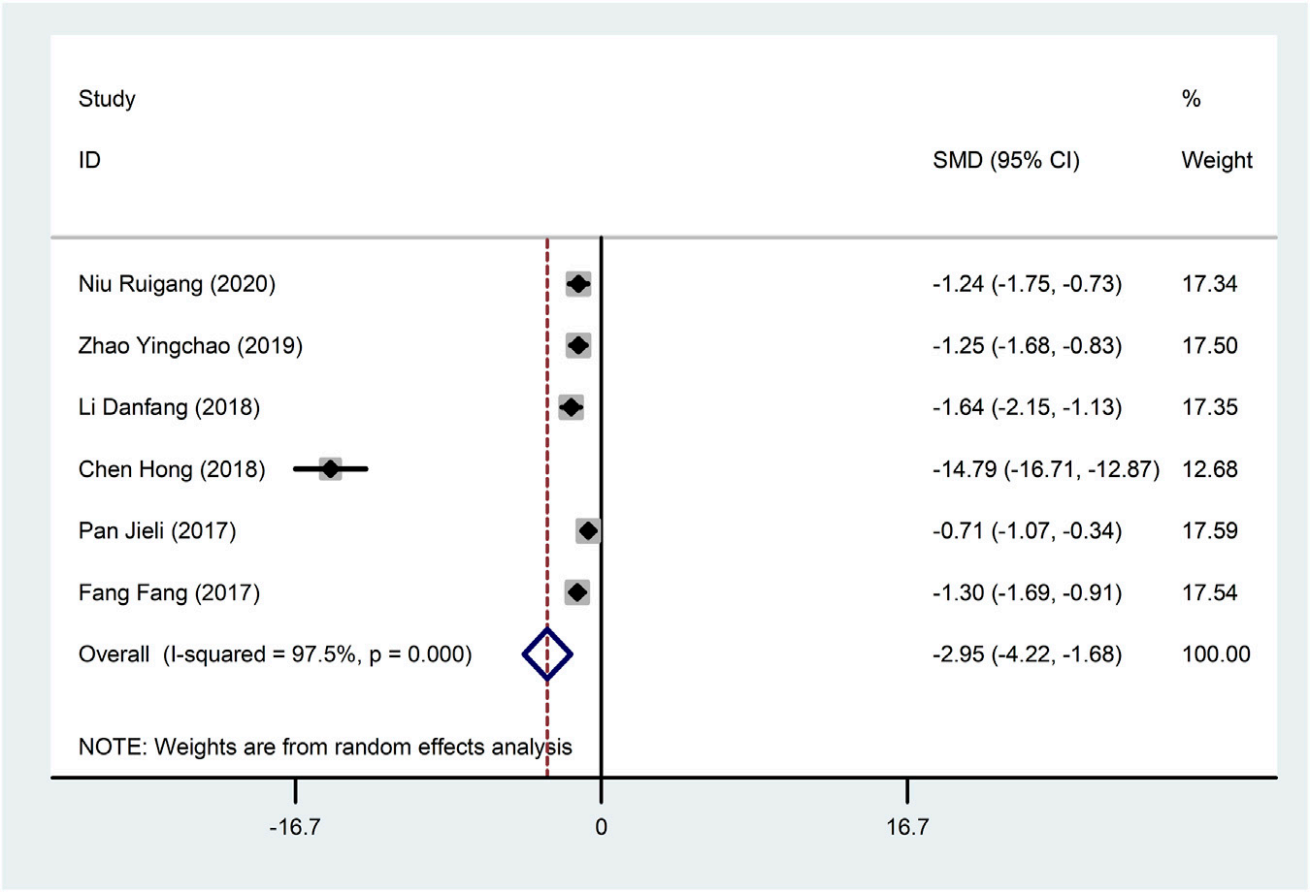
Forest plot of IL-6.

Six studies (Fang et al., 2017; Pan et al., 2017; Li, 2018; Hong et al., 2018; Zhao et al., 2019; Niu, 2020) reported IL-6 with large heterogeneity (I^2^ = 97.5%), so meta-analysis using a random effects model showed statistically significant differences (SMD = −2.95, 95%CI (−4.22, −1.68), *p* < 0.0001), which indicated that CDDP added to CWT was superior to CWT in reducing IL-6 (Figure 10). To further clarify the sources of heterogeneity, we also conducted meta-regression to evaluate the effects of the publication time (Coed. = −3.463, *p* = 0.720), sample size (Coed. = 3.463, *p* = 0.720), and treatment duration (Coed. = −4.597, *p* = 0.617), the results did not reveal the source of heterogeneity among studies (Table 2). Subgroup analyses showed better homogeneity in subgroups with sample sizes less than 50 cases (Table 3, Supplementary Material S2).

#### 4.3.3 TNF-α

**FIGURE 11:**
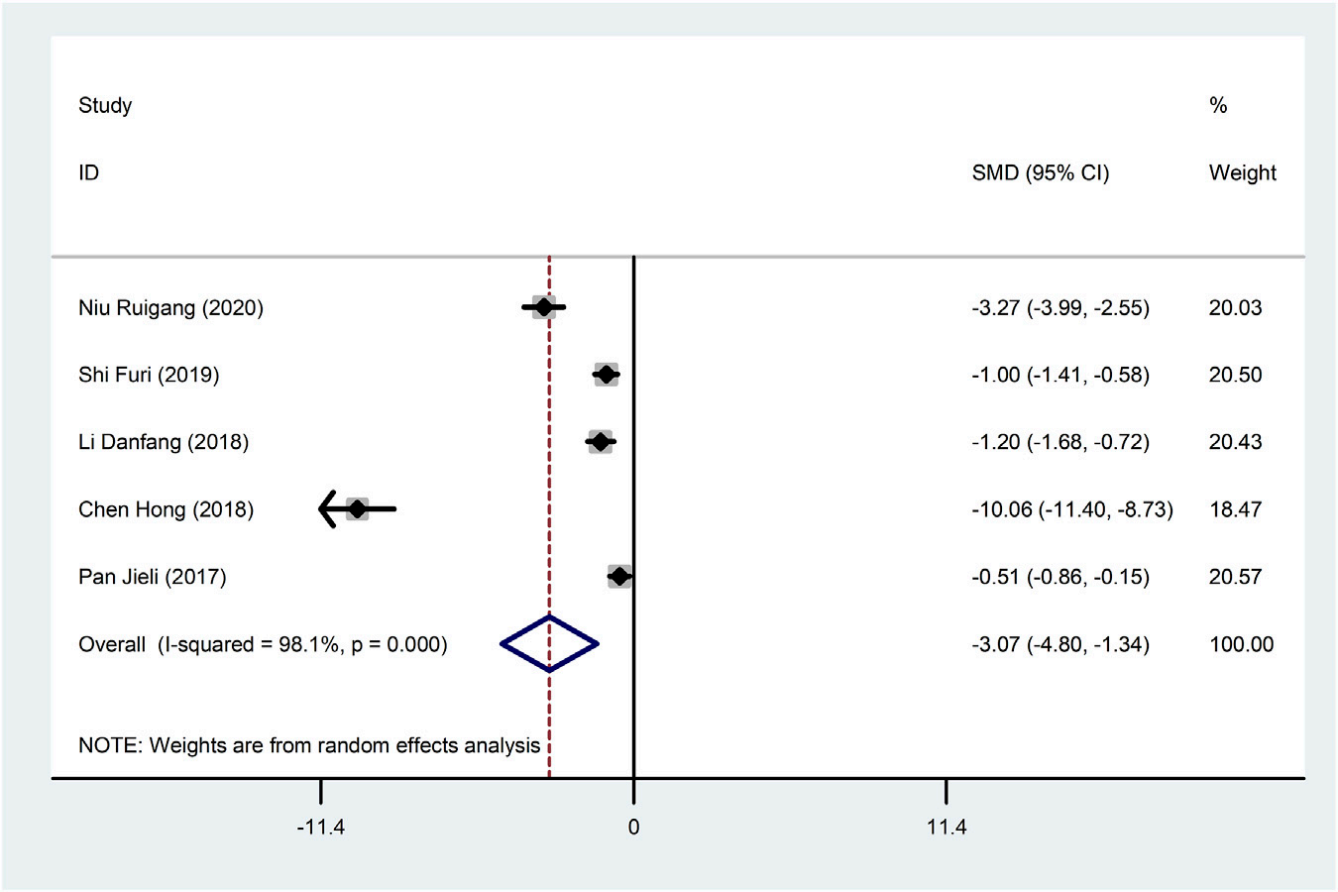
Forest plot of TNF-α

Five studies (Pan et al., 2017; Li, 2018; Hong et al., 2018; Shi, 2019; Niu, 2020) reported TNF-α with large heterogeneity (I^2^ = 98.1%), so meta-analysis using a random effects model showed statistically significant differences (SMD = −3.07, 95%CI (−4.80, −1.34), *p* = 0.001), which indicated that CDDP added to CWT was superior to CWT in reducing TNF-α (Figure 11). To further clarify the sources of heterogeneity, we also conducted meta-regression to evaluate the effects of the publication time (Coed. = −3.342, *p* = 0.529) and sample size (Coed. = −1.577, *p* = 0.724), the results did not reveal the source of heterogeneity among studies (Table 2). The results of the subgroup analysis failed to identify sources of heterogeneity (Table 3).

### 4.4 Adverse reactions/adverse events

**FIGURE 12:**
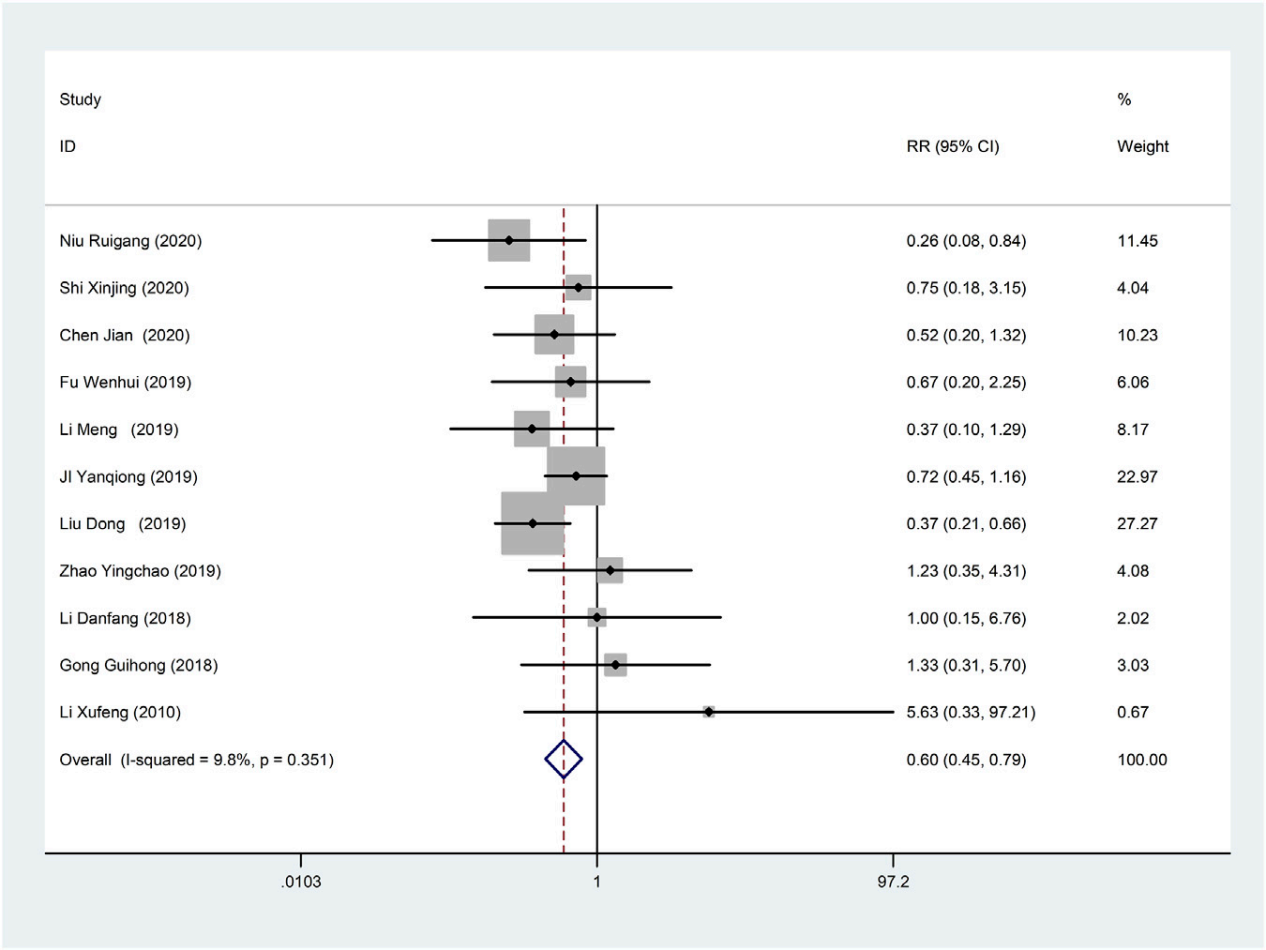
Forest plot of Adverse Reactions/Adverse Events.

Eleven studies (Li et al., 2010; Li, 2018; Guihong et al., 2018; Ji et al., 2019; Li et al., 2019; Liu and Zhang, 2019; Wenhui, 2019; Zhao et al., 2019; Jian, 2020; Niu, 2020; Shi, 2020) reported adverse reactions/adverse events with fine homogeneity, so meta-analysis using fixed-effects model showed statistically significant between the two groups (RR = 0.60, 95% CI (0.45, 0.79), *p* < 0.0001), suggesting that CDDP added to CWT was superior to CWT (Figure 12). The incidence of adverse reactions/adverse events was 11.6% (62/535) in the treatment group and 18.6% (96/515) in the control group.

### 4.5 Results of publication bias assess

**FIGURE 13:**
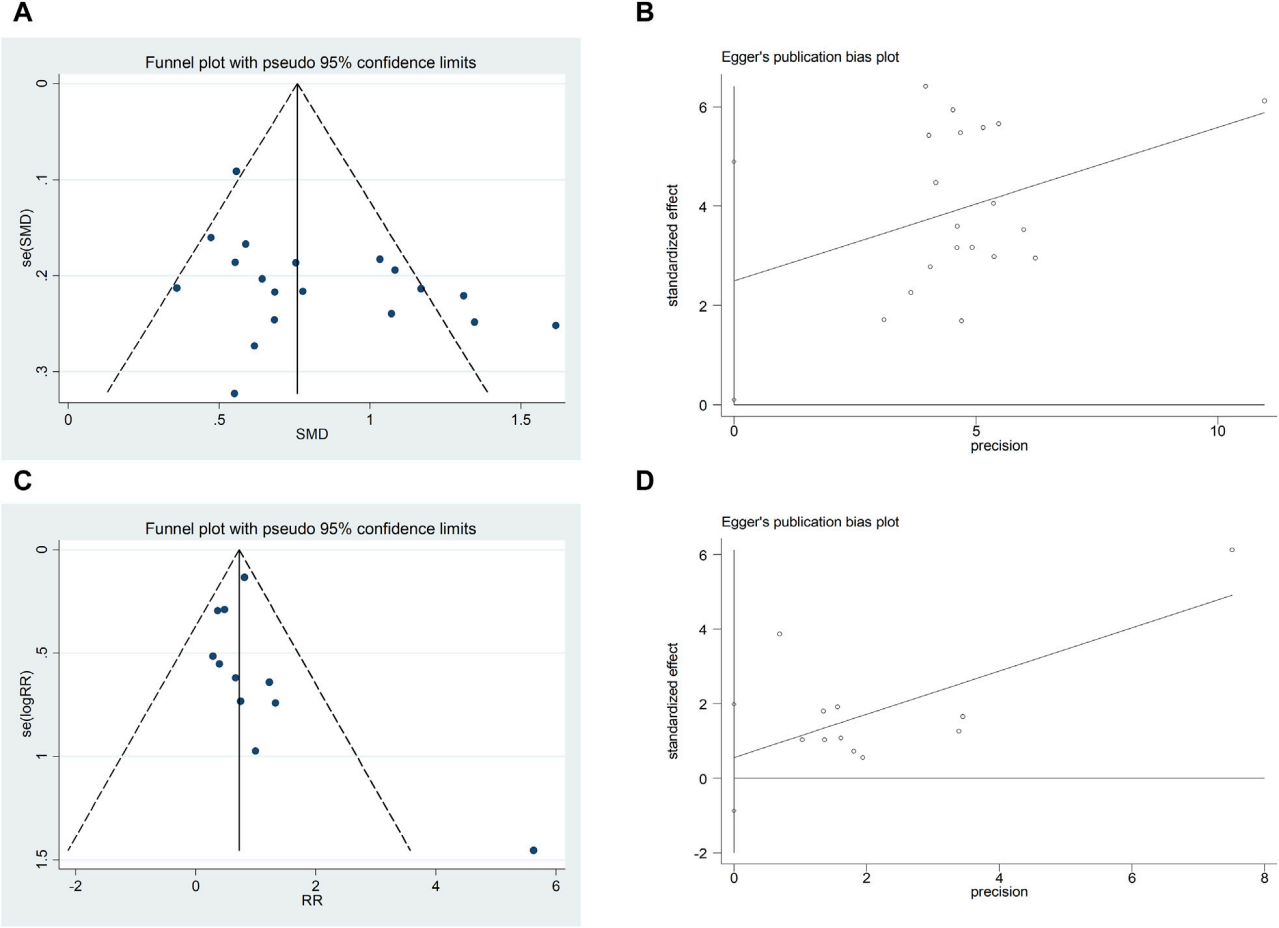
A-B Funnel plot and Egger’s test for LVEF; C-D Funnel plot and Egger’s test for adverse reaction/adverse event.

We assessed publication bias for metrics that included more than 10 studies. The LVEF funnel plot exhibited left-right asymmetric distribution characteristics, indicating potential publication bias. The quantitative results of Egger’s test are consistent with the qualitative results of the funnel plot (t = 2.19, 95%CI, 0.096 to 4.895, *p* = 0.042) (Figures 13A,B). It may be related to the quality and sample size of the included studies, and selective reporting cannot be ruled out due to lack of information on clinical trial registration or study protocols. The adverse reaction/adverse event funnel plot exhibited left-right symmetry, and the quantitative results of the egger’s test were consistent with the qualitative results of the funnel plot (t = −2.13, 95%CI, −397.27 to−2.28, *p* = 0.048) (Figures 13C,D).

### 4.6 GRADE evidence evaluation

The GRADE evaluation tool was used to grade the evidence for each study’s outcome indicators, taking into account the risk of bias, inconsistency of results, directness of evidence, precision of evidence, and publication bias. Downgrading the quality of the evidence when one or more factors are present in the results of each study. TCER was evaluated as moderate. LVEF, LVEDD, IL-6, TNF-α were evaluated as low. hs-CRP, CK-MB, cTnT, and NT-proBNP were evaluated as very low because of the risk of bias associated with small sample sizes for these indicators (Table 4).

**TABLE 4:** GRADE Evidence evaluation.

Quality assessment							Summary of findings				Importance
							No of patients		Effect	Quality	
No of studies	Design	Limitations	Inconsistency	Indirectness	Imprecision	Other considerations	CDDP + CWT	CWT	Relative		
									(95% CI)		
LVEF											
19	randomised trials	serious^1^	serious^2^	no serious indirectness	no serious imprecision	none	1,164	1,142	SMD 0.82 higher (0.67–0.97 higher)	⊕⊕ΟΟ	CRITICAL
										LOW	
LVEDD											
9	randomised trials	serious^1^	serious^2^	no serious indirectness	no serious imprecision	none	537	538	SMD 1.00 lower (1.27–0.73 lower)	⊕⊕ΟΟ	IMPORTANT
										LOW	
NT-proBNP											
3	randomised trials	serious^1^	serious^2^	no serious indirectness	serious^3^	none	147	147	SMD 3.83 lower (6.34–1.31 lower)	⊕ΟΟΟ	IMPORTANT
										VERY LOW	
cTnT											
2	randomised trials	serious^1^	serious^2^	no serious indirectness	serious^3^	none	120	122	SMD 1.16 lower (2.3–0.02 lower)	⊕ΟΟΟ	IMPORTANT
										VERY LOW	
CK-MB											
3	randomised trials	serious^1^	serious^2^	no serious indirectness	serious^3^	none	164	165	SMD 1.07 lower (1.88–0.27 lower)	⊕ΟΟΟ	IMPORTANT
										VERY LOW	
TCER											
6	randomised trials	serious^1^	no serious inconsistency	no serious indirectness	no serious imprecision	none	249/259 (96.1%)	212/258 (82.2%)	RR 1.17 (1.1–1.24)	⊕⊕⊕Ο	CRITICAL
										MODERATE	
hs-CRP											
4	randomised trials	serious^1^	serious^2^	no serious indirectness	serious^3^	none	177	176	SMD 1.50 lower (1.99–1.01 lower)	⊕ΟΟΟ	IMPORTANT
										VERY LOW	
IL-6											
6	randomised trials	serious^1^	serious^2^	no serious indirectness	no serious imprecision	none	313	304	SMD 2.95 lower (4.22–1.68 lower)	⊕⊕ΟΟ	IMPORTANT
										LOW	
TNF-α											
5	randomised trials	serious^1^	serious^2^	no serious indirectness	no serious imprecision	none	251	243	SMD 3.07 lower (4.8–1.34 lower)	⊕⊕ΟΟ	IMPORTANT
										LOW	

Note: ^1^the included studies have certain defects in randomization, allocation concealment, and blinding; ^2^ the included studies are highly heterogeneous; ^3^ Relatively few patients were included.

### 4.7 Results of TSA

**FIGURE 14:**
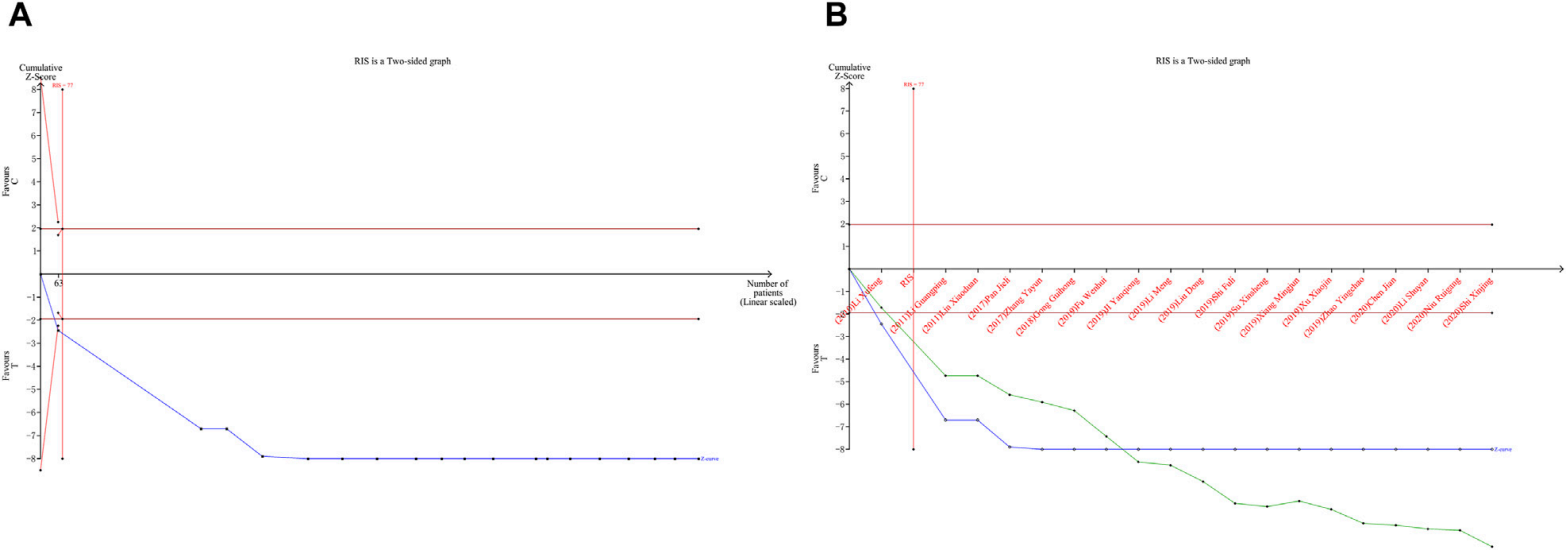
A Trial sequential analysis of LVEF; B penalty statistics analysis.

TSA was performed on LVEF. Type I error was defined as 5%, the information axis was set to a cumulative sample size, statistical efficacy was 80%, and the cumulative Z-value crossed the traditional and TSA bounds after item 2 to obtain a positive conclusion in advance, using the sample size as the required information size (RIS). The penalized curve exceeded the traditional bound of Z = 1.96, further confirming the clinical efficacy of CDDP in improving LVEF (Figures 14A,B).

## 5 Discussion

Early reperfusion is a typical therapy for AMI that can effectively restore blood flow to ischemic myocardial tissue. However, reperfusion itself will increase irreversible damage to coronary artery circulation and accelerate and expand the IRI (Qi et al., 2021). One of the ways in which TCM has made an outstanding contribution to the development of medicine in the world is through the availability of effective natural compounds. TCM is characterised by the adjustment of multiple components and multiple targets to transform the organism from an abnormal to a normal state (Yin-Yang balance) (Wang et al., 2018).

### 5.1 From bench to bedside

CDDP is a highly dispersed state of Chinese patent medicine made of *Salviae Miltiorrhizae Radix et Rhizoma*, *Notoginseng Radix*, and *Borneolum Syntheticum* as the main ingredients and processed by modern technology, which has the effect of activating blood and activating stasis, regulating qi and relieving pain. The inflammatory response induced after PCI promotes an excessive increase in oxygen free radicals, which triggers ventricular remodeling and myocardial injury and is an initiating factor for thrombus re-formation (Li et al., 2023). Pharmacological studies have found CDDP to have coronary artery dilation, anti-inflammatory, anti-thrombotic, and vascular endothelial and cardiomyocyte protection effects, thereby further reversing IRI (Bei et al., 2017). Panax notoginseng (PNS), an active ingredient in *Notoginseng Radix*, can effectively inhibit oxygen sugar deprivation-induced apoptosis, probably by inhibiting oxidative stress and inducing Akt phosphorylation (Chen et al., 2011). PNS also reduces the expression of pro-inflammatory factors by inhibiting the RAGE, MAPK signaling pathway in apoE (−/−) mouse lesions (Dou et al., 2012). Ginsenosides Rb1 and Rg1 activate nitric oxide (NO) and promote endothelium-dependent vasodilatation by regulating endothelial cell PI3K/Akt/eNOS pathway and L-arginine transport (Pan et al., 2012). Ginsenoside Rd prevents the development of atherosclerosis by inhibiting Ca^2+^ inward flow through voltage-independent Ca^2+^ channels (Li et al., 2011). CDDP has a wide range of applications in cardiovascular disease, and most RCTs have demonstrated significant clinical efficacy of CDDP-assisted therapy in AMI undergoing PCI. However, trials with large sample sizes are lacking. This meta-analysis provides a valid evidence-based rationale for the clinical use of CDDP in the treatment of AMI undergoing PCI.

### 5.2 Robustness of meta-analysis and credibility of evidence

Meta-analysis results showed that CDDP has good positive utility for AMI undergoing PCI treatment, can reduce hs-CRP, TNF-alpha, IL-6 levels, inhibit myocardial local inflammatory response, improve myocardial injury indexes such as CK-MB, NT-proBNP, cTnT, improve LVEF, reduce LVEDD, and at the same time, it shows strong advantages in reducing the incidence of adverse reactions/adverse events. Due to the low heterogeneity, we can clarify that CDDP combined with CWT is superior to CWT in improving TCER. Interestingly despite our choice of randomized models for data statistics, sensitivity analyses, meta-regression analyses, and subgroup analyses to eliminate heterogeneity between studies, a small number of outcome metrics remained highly heterogeneous, prompting us to be cautious in interpreting the results in clinical practice. GRADE results showed that the quality of evidence ratings for most outcome indicators were very low and low. Currently, a number of studies are rated as low quality due to methodological limitations, mainly due to risk of bias, inconsistent results, indirect evidence. Most studies lacked blinding and allocation concealment designs during randomization. However, the emerging view is that unblinded pragmatic trials should be recommended because they emphasize practical applicability and extrapolation to improve the external validity of real-world trials rather than treatment effects, even though this approach is a major contributor to the low quality of included studies (Sox and Lewis, 2016). TSA was performed to exclude possible false-positive results in order to improve the robustness of the results of the meta-analysis. The results of the TSA showed that the total number of samples collected in this study met the requirements of the meta-analysis, which excluded the possibility of false-positive results, and further confirmed the efficacy of the CDDP for the treatment of AMI undergoing PCI.

### 5.3 Limitations and perspectives

The purpose of this study is to summarize and evaluate the efficacy and safety of CDDP for the treatment of AMI undergoing PCI according to the updated and optimized methods of PRISMA, so that the results are sufficiently comparable and convincing. Nevertheless, despite the encouraging results, there are inevitable limitations to this study: 1) The overall quality of the studies was poor, with most being single-center, small-sample trials and with a lack of uniformity in reporting methods for outcome indicators. The poor quality of some of the studies resulted in high risk and heterogeneity between studies, which may reduce the robustness of the results of meta-analysis. 2) As original studies related to most of the outcome metrics were scarce, only studies on LVEF improvement were assessed for publication bias. 3) In addition to the indicators analyzed in this study, there are individual studies that reported TCM efficacy scores, triglycerides (TG), and total cholesterol (TC), etc. However, due to the small number of studies and the inconsistency in the way the outcome indicators were measured, this study did not perform a combined analysis, which is to be enriched and improved in the future. 4) The evaluation of drug efficacy in the related field requires a long period of time to be completed due to the lengthy process of further treatment after PCI. The study’s inclusion of a short course of RCT and the lack of long-term follow-up means that the drug’s long-term efficacy is not yet known. 5) There were few studies that explicitly stated that they conducted their RCTs in accordance with the Consolidated Standards of Reporting Trials (CONSORT), and most did not provide registry information. 6) The lack of relevant information on blinding and allocation concealment in a small number of studies may lead to an exaggerated effect of the results.

In the future, clinical RCTs on the adjuvant treatments of CDDP against AMI should be improved in the following aspects. First, at the design stage, protocols for RCTs should comply with the CONSORT statement. High-quality clinical data conforming to international standards such as the CONSORT statement is absolutely essential for the further development and acceptance of TCM, and the improvement of the quality of clinical data will be a key step in the future of TCM. Second, RCTs should use a rigorous blinded design with recognized and uniform outcomes to evaluate the therapeutic efficacy of CDDP. Third, RCTs should perform sample size estimation and be designed with a sufficiently long follow-up period for evaluation. Fourth, improvements in TCM symptoms should be recorded in detail to provide evidence for future TCM consultations and treatments to better improve quality of life.

## 6 Conclusion

In conclusion, adjuvant treatment of AMI with CDDP has shown exciting and safe benefits in improving cardiac function and reducing inflammatory response in patients with AMI undergoing PCI. Nevertheless, due to the poor quality of some of the included studies, it needs to use RCTs with rigorous designs and long follow-up periods to confirm and update the results.

## Data Availability

The original contributions presented in the study are included in the article/Supplementary Material, further inquiries can be directed to the corresponding author.

## Abbreviations

AMI, acute myocardial infarction; CDDP, compound danshen drip pill; STEMI, ST-segment elevation myocardial infarction; NSTEMI, Non-ST-segment elevation myocardial infarction; PCI, percutaneous coronary intervention; GRADE, grading of recommendations assessment, development and evaluation; TSA, trial sequential analysis; TCM, traditional Chinese medicine; CWT, conventional western treatment; ACEI, angiotensin-converting enzyme inhibitors; ARB, angiotensin receptor blockers; LVEF, left ventricular ejection fraction; TCER, total clinical effective rate; NT-proBNP, N-terminal pro-B-type natriuretic peptide; hs-CRP, high-sensitivity C-reactive protein; IL-6, interleukin-6; TNF-α, tumor necrosis factor-alpha; CK-MB, creatine kinase isoenzyme; IRI, ischemia reperfusion injury; PNS, panax notoginseng; NO, nitric oxide; CONSORT, consolidated standards of reporting trials.
