# Corrigendum: Effect of adjuvant therapy with compound danshen drip pill on inflammatory factors and cardiac function after percutaneous coronary intervention for acute myocardial infarction: a systematic review and meta-analysis

**DOI:** 10.3389/fphar.2024.1470318

**Published:** 2024-08-06

**Authors:** Genhao Fan, Menglin Liu, Huanhuan Song, Yongxia Wang

**Affiliations:** ^1^ The First Affiliated Hospital of Zhengzhou University, Zhengzhou, Henan, China; ^2^ The First Affiliated Hospital of Henan University of Chinese Medicine, Zhengzhou, Henan, China

**Keywords:** Acute myocardial infarction, Compound danshen drip pill, Meta-analysis, Systematic review, Cardiac function

In the published article, there was an error in **Affiliation 1**. Instead of “Henan Provincial Chest Hospital, Zhengzhou, Henan, China,” it should be “The First Affiliated Hospital of Zhengzhou University, Zhengzhou, Henan, China.”

The authors apologize for this error and state that this does not change the scientific conclusions of the article in any way. The original article has been updated.

