# Supplementary material for: Effect of adjuvant therapy with compound danshen drip pill on inflammatory factors and cardiac function after percutaneous coronary intervention for acute myocardial infarction: a systematic review and meta-analysis
Source: Front Pharmacol. 2024 Apr 16;15:1345897. doi: 10.3389/fphar.2024.1345897 (PMC11058228; doi:10.3389/fphar.2024.1345897)

LVEF Subgroup analyses

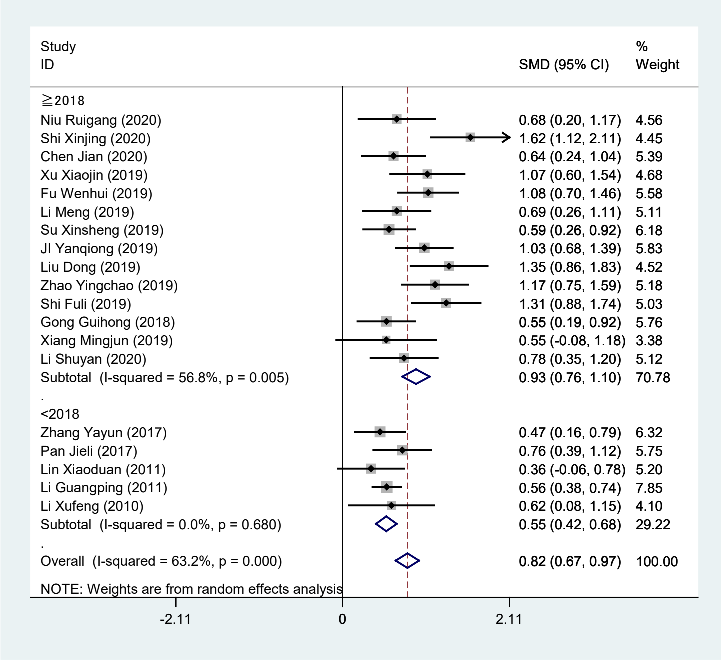

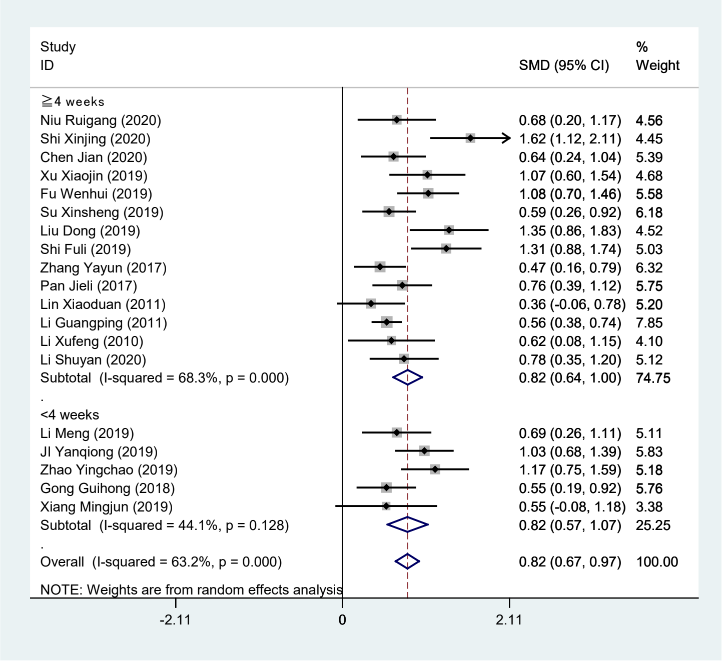

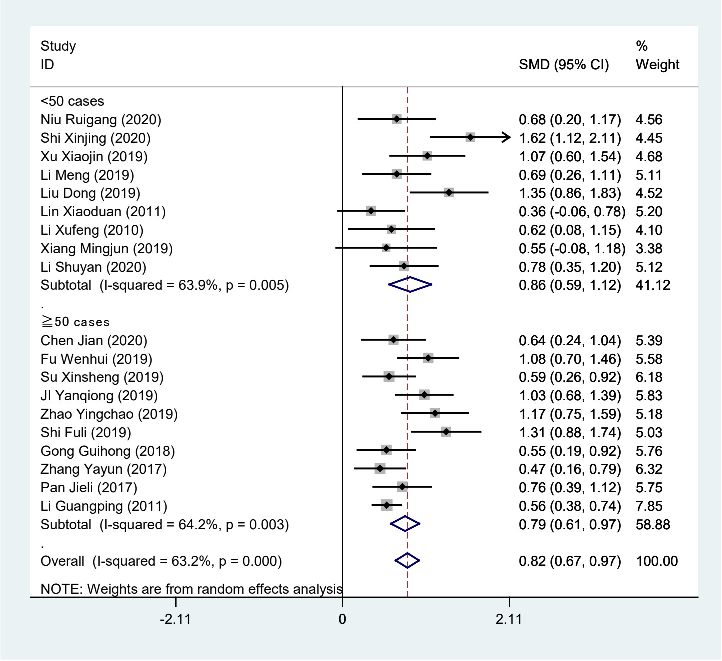

NT-proBNP Subgroup analyses

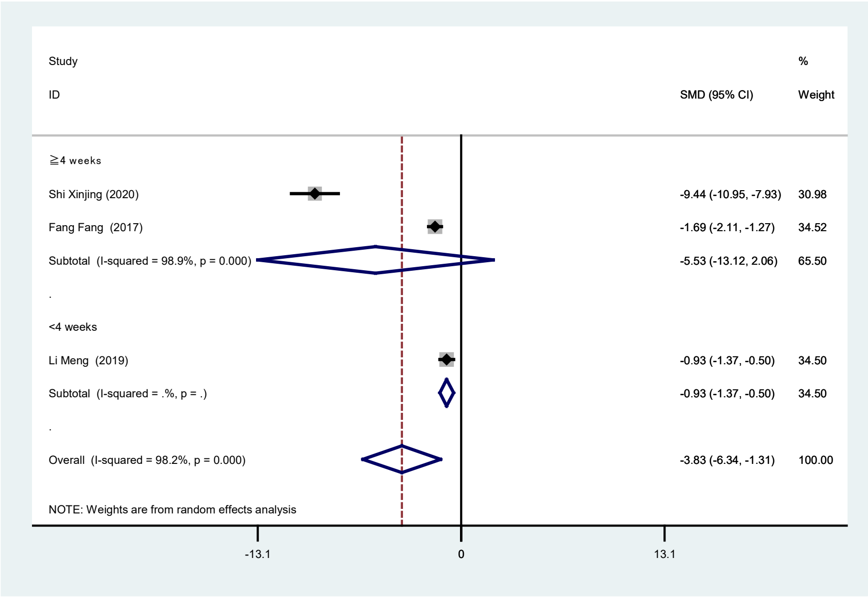

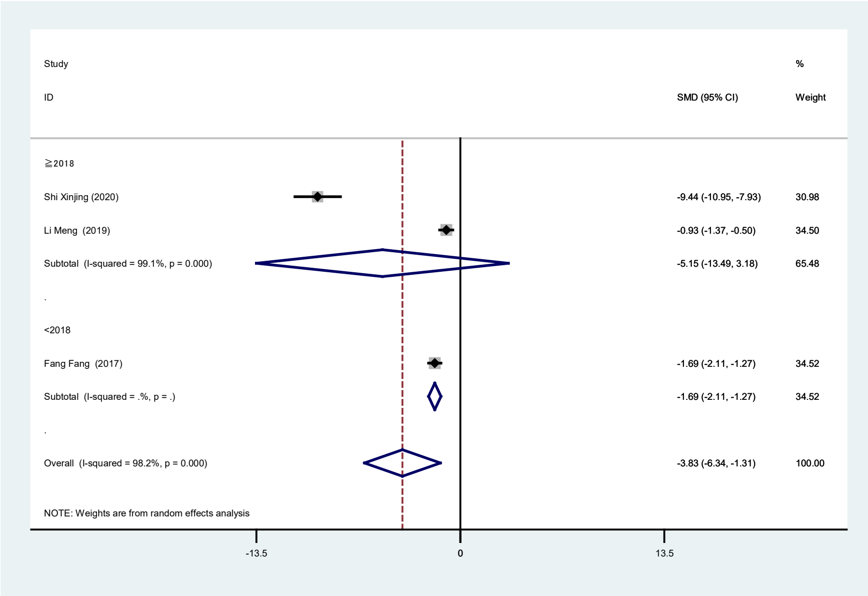

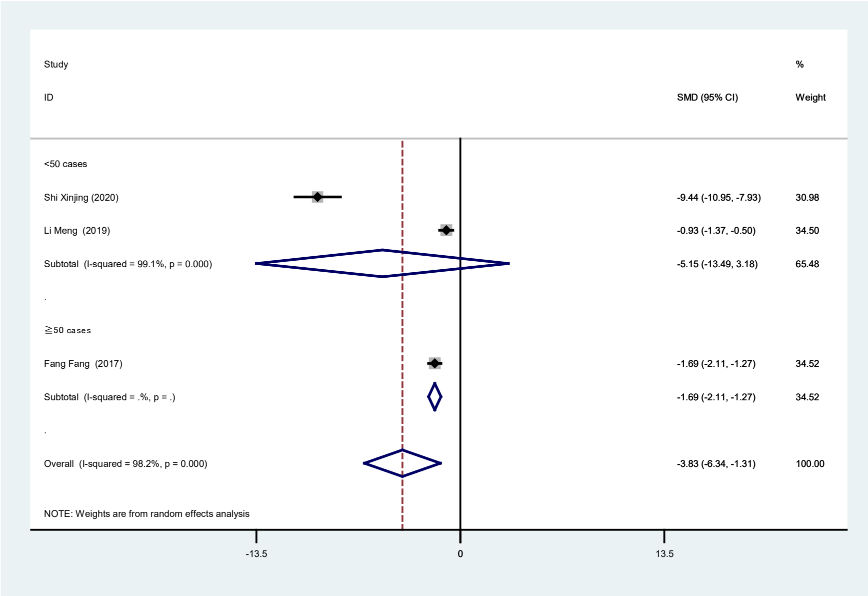

hs-CRP Subgroup analyses

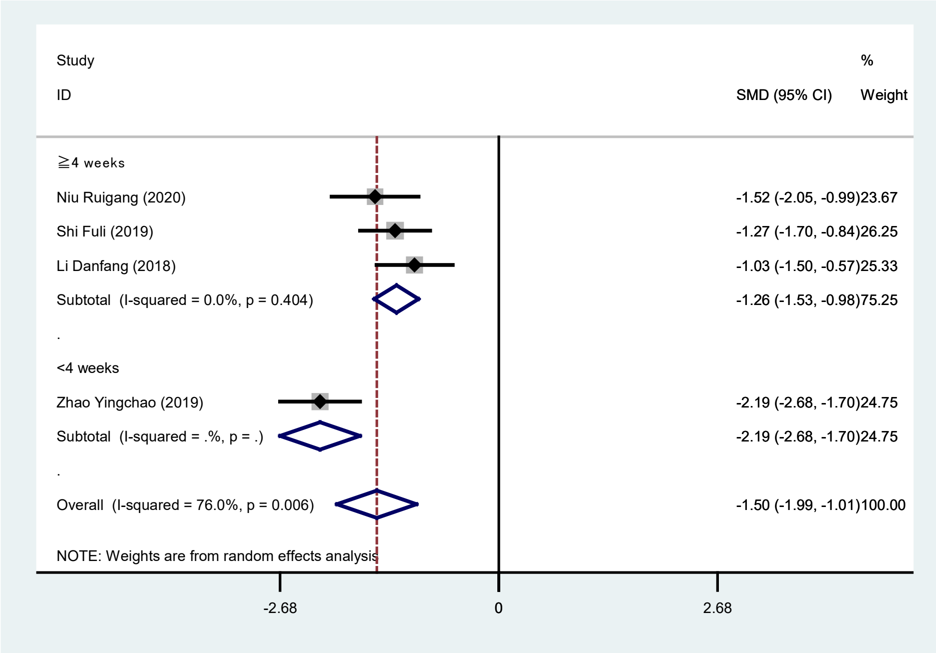

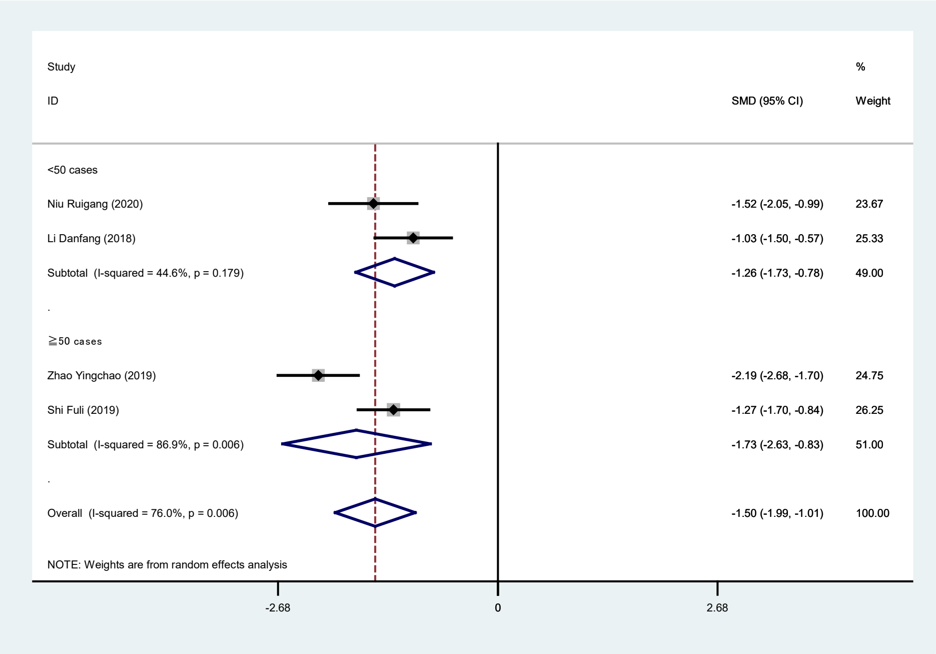

IL-6 Subgroup analyses

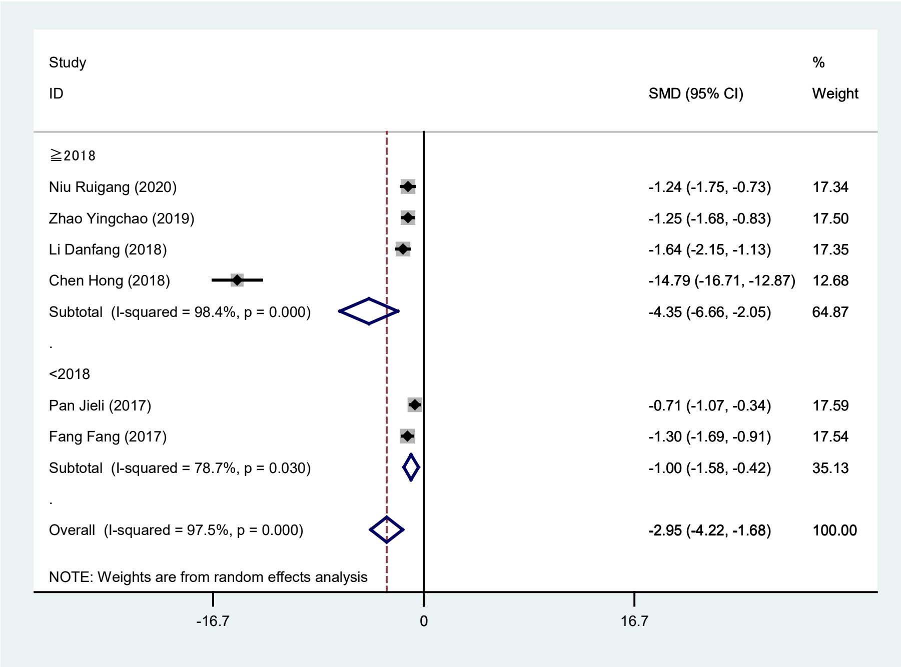

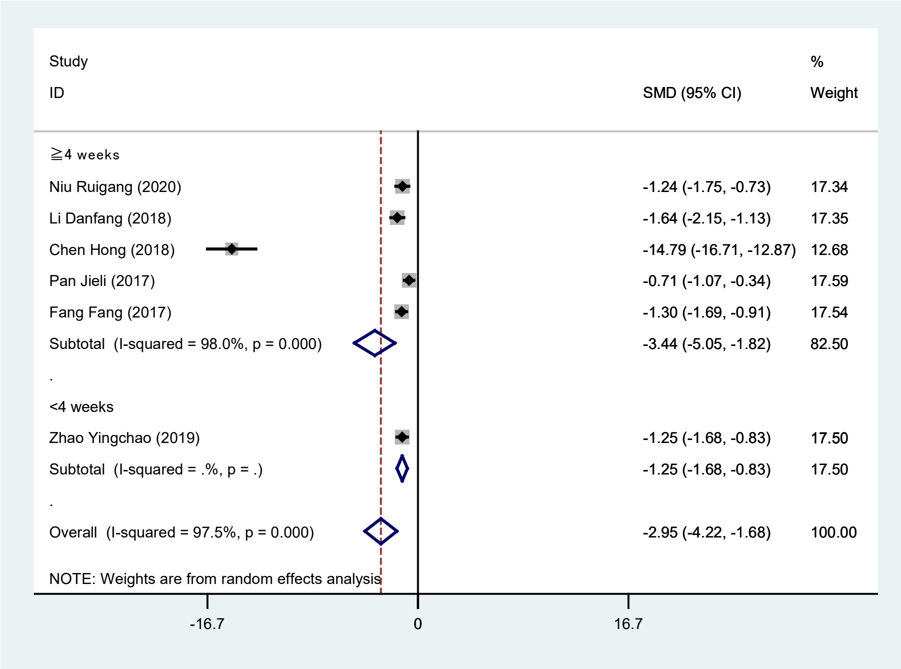

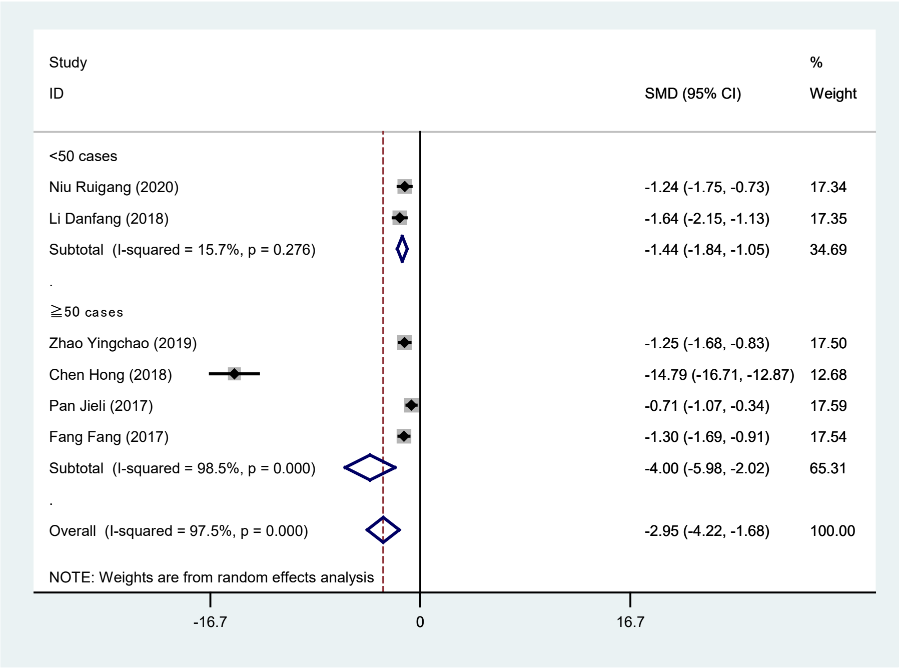

TNF-α Subgroup analyses

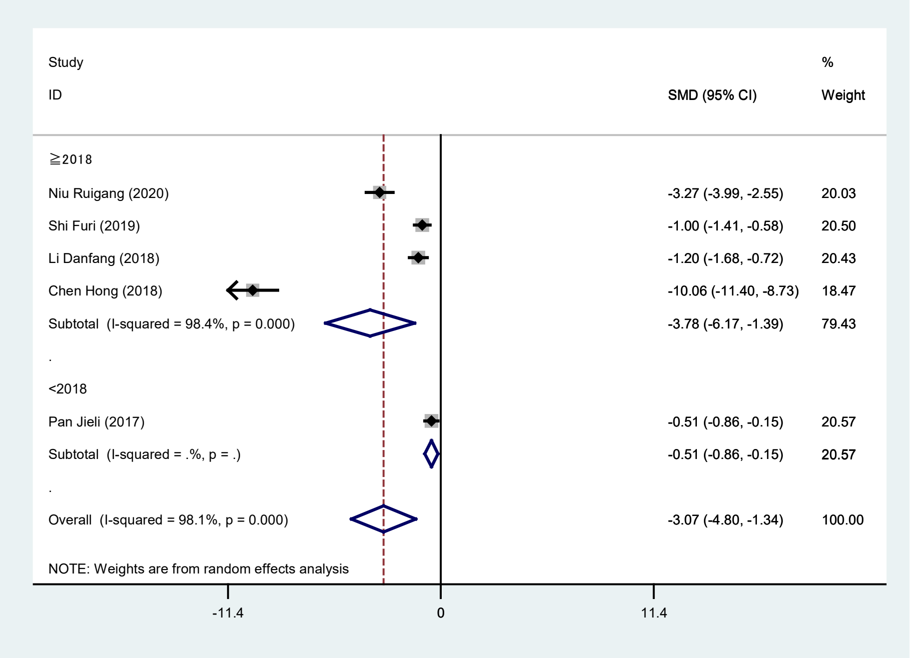

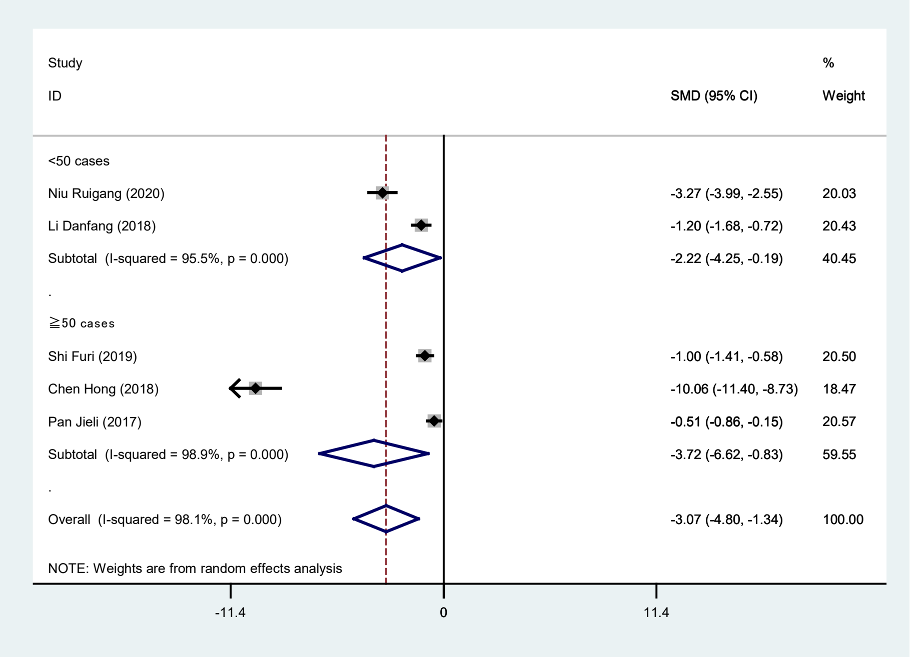

LVEDD Subgroup analyses

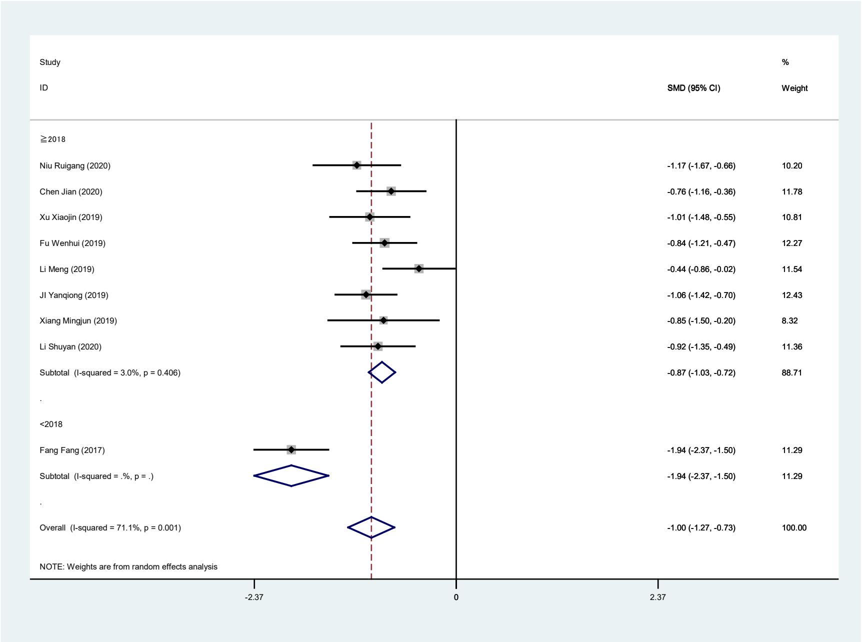

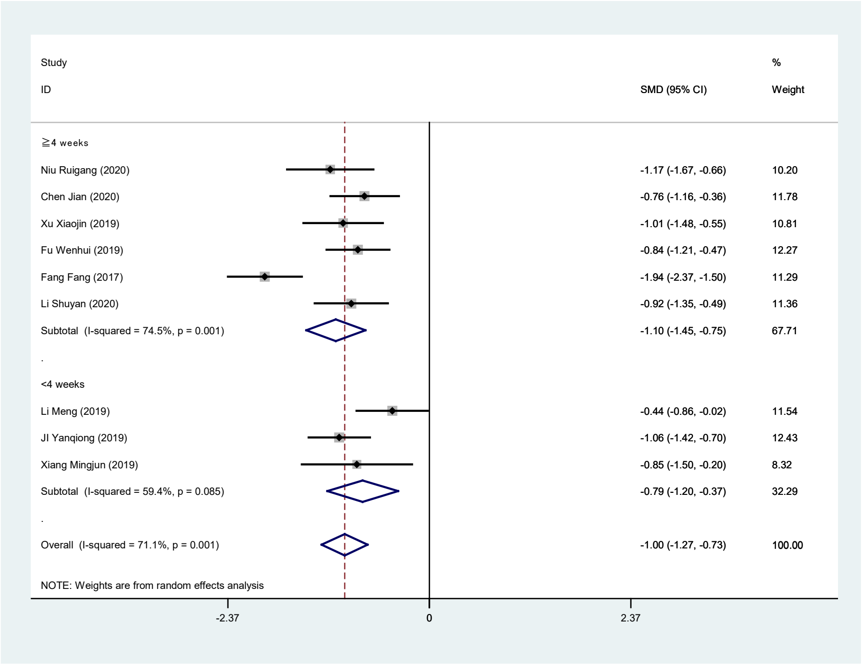

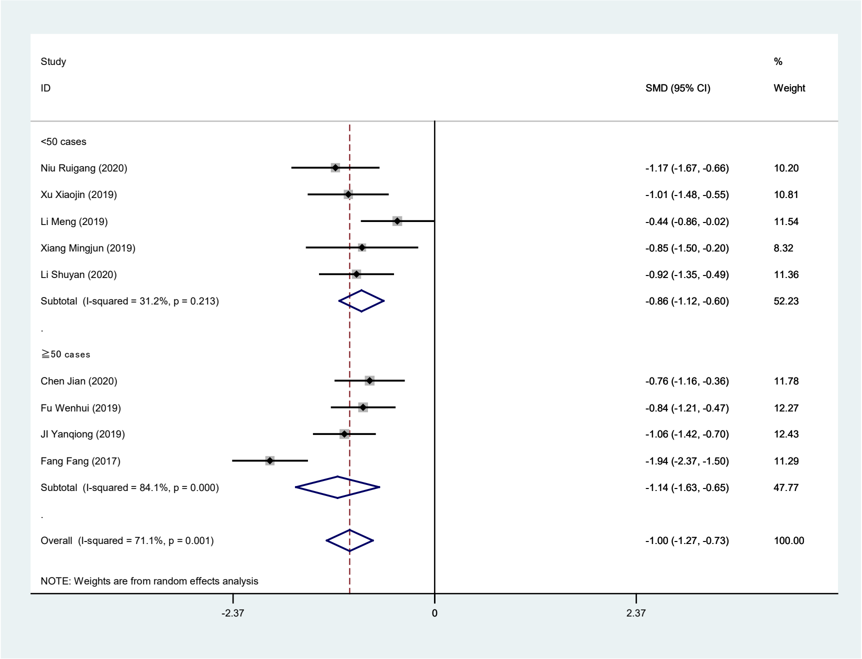

CK-MB Subgroup analyses

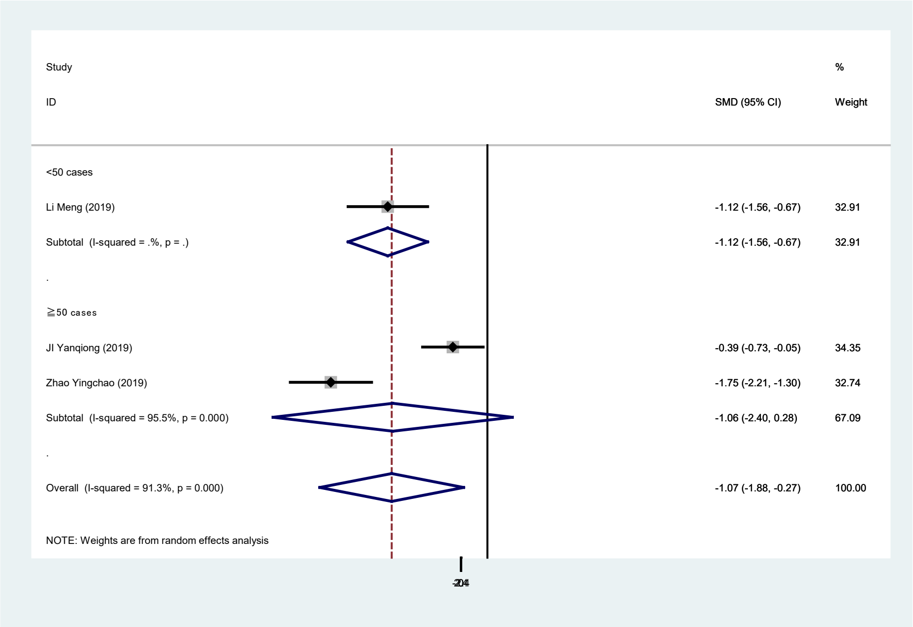

Supplement: Supplementary file 2 [file DataSheet1.PDF]
