# Supplementary material for: Effect of adjuvant therapy with compound danshen drip pill on inflammatory factors and cardiac function after percutaneous coronary intervention for acute myocardial infarction: a systematic review and meta-analysis
Source: Front Pharmacol. 2024 Apr 16;15:1345897. doi: 10.3389/fphar.2024.1345897 (PMC11058228; doi:10.3389/fphar.2024.1345897)
Supplement: Supplementary file 3 [file DataSheet2.docx]

# Supplementary File S2. Search strategies for databases.

| Number | Search terms |
| --- | --- |
|  | **Pubmed** |
| #1 | ("Acute Myocardial Infarction"[MeSH Terms] OR " Percutaneous Coronary Intervention"[Title/Abstract] OR "AMI" OR "PCI") |
| #2 | ("Compound Danshen Dropping Pill"[Title/Abstract] OR " Danshen Dropping Pill"[Title/Abstract]) |
| #3 | "randomized controlled trial"[Title/Abstract] OR "randomised controlled trial"[Title/Abstract] OR "randomized"[Title/Abstract] OR"randomised"[Title/Abstract] OR "RCT"[Title/Abstract] |
| #4 | #1 and #2 and #3 |
|  | **Cochrane Library** |
| #1 | (Acute Myocardial Infarction [Title/Abstract/keywords]) OR (Percutaneous Coronary Intervention [Title/Abstract/keywords]) |
| #2 | (Compound Danshen Dropping Pill [Title/Abstract/keyword]) OR (Danshen Dropping Pill [Title/Abstract/keyword]) |
| #3 | (randomized controlled trial [Title/Abstract]) OR (randomised controlled trial [Title/Abstract]) OR (randomized) OR (randomised [Title/Abstract]) OR (RCT[Title/Abstract]) |
| #4 | #1 and #2 and #3 |
|  | **Web of Science** |
| #1 | (TS = Acute Myocardial Infarction OR Percutaneous Coronary Intervention) |
| #2 | (TS = Compound Danshen Dropping Pill OR Danshen Dropping Pill) |
| #3 | (TS = randomized controlled trial OR randomised controlled trial OR randomized OR randomised OR RCT) |
| #4 | #1 and #2 and #3 |
|  | **Embase** |
| #1 | (Acute Myocardial Infarction [Title/Abstract/keywords]) OR (Percutaneous Coronary Intervention [Title/Abstract/keywords]) |
| #2 | (Compound Danshen Dropping Pill [Title/Abstract/keyword]) OR Danshen Dropping Pill [Title/Abstract/keyword]) |
| #3 | (randomized controlled trial [Title/Abstract]) OR (randomised controlled trial [Title/Abstract]) OR (randomized [Title/Abstract]) OR (randomized [Title/Abstract]) OR (RCT[Title/Abstract]) |
| #4 | #1 and #2 and #3 |
| **China National Knowledge Infrastructure (CNKI)** | |
| (主题=复方丹参滴丸 + 丹参滴丸)  AND  (主题=急性心肌梗死 + 经皮冠状动脉介入治疗)  AND  (摘要=随机对照 + 随机 + 试验 + RCT) | |
| **China Science and Technology Journal Database (VIP)** | |
| (题名或关键词=复方丹参滴丸 + 丹参滴丸)  AND  (题名或关键词=急性心肌梗死 + 经皮冠状动脉介入治疗)  AND  (摘要=随机对照 + 随机 + 试验 + RCT) | |
| **Wanfang Database (Wangfang)** | |
| (题名或关键词=复方丹参滴丸OR丹参滴丸)  AND  (题名或关键词=急性心肌梗死 OR经皮冠状动脉介入治疗)  AND  (摘要=随机对照 OR 随机 OR 试验 OR RCT) | |
| **Chinese Biomedical Literature Database (CBM)** | |
| ("复方丹参滴丸"[标题:智能] OR "丹参滴丸"[标题:智能])  AND  ( "急性心肌梗死"[标题:智能] OR "经皮冠状动脉介入治疗"[标题:智能])  AND  ("随机对照"[摘要:智能] OR "随机"[摘要:智能] OR "试验"[摘要:智能] OR "RCT"[摘要:智能] | |
